# Association between HIV infection and hypertension: a global systematic review and meta-analysis of cross-sectional studies

**DOI:** 10.1186/s12916-021-01978-7

**Published:** 2021-05-13

**Authors:** Katherine Davis, Pablo Perez-Guzman, Annika Hoyer, Ralph Brinks, Edward Gregg, Keri N. Althoff, Amy C. Justice, Peter Reiss, Simon Gregson, Mikaela Smit

**Affiliations:** 1 MRC Centre for Global Infectious Disease Analysis, Department of Infectious Disease Epidemiology, St Mary’s Campus, Imperial College London, London, W2 1PG UK; 2 Department of Statistics, Ludwig-Maximilians-University Munich, Munich, Germany; 3 Hiller Research Unit of Rheumatology, University Hospital Duesseldorf, Duesseldorf, Germany; 4 Department of Epidemiology and Biostatistics, Imperial College London, London, UK; 5 Department of Epidemiology, Johns Hopkins University, Baltimore, MD USA; 6 Schools of Medicine and Public Health, Yale University, New Haven, CT USA; 7 Department of Global Health, Amsterdam University Medical Centers, University of Amsterdam and Amsterdam Institute for Global Health and Development, Amsterdam, Netherlands; 8 HIV Monitoring Foundation, Amsterdam, Netherlands; 9 Biomedical Research and Training Institute, Harare, Zimbabwe

**Keywords:** HIV, Hypertension, Blood pressure, Systematic review, Meta-analysis

## Abstract

**Background:**

Improved access to effective antiretroviral therapy has meant that people living with HIV (PLHIV) are surviving to older ages. However, PLHIV may be ageing differently to HIV-negative individuals, with dissimilar burdens of non-communicable diseases, such as hypertension. While some observational studies have reported a higher risk of prevalent hypertension among PLHIV compared to HIV-negative individuals, others have found a reduced burden. To clarify the relationship between HIV and hypertension, we identified observational studies and pooled their results to assess whether there is a difference in hypertension risk by HIV status.

**Methods:**

We performed a global systematic review and meta-analysis of published cross-sectional studies that examined hypertension risk by HIV status among adults aged > 15 (PROSPERO: CRD42019151359). We searched MEDLINE, EMBASE, Global Health and Cochrane CENTRAL to August 23, 2020, and checked reference lists of included articles. Our main outcome was the risk ratio for prevalent hypertension in PLHIV compared to HIV-negative individuals. Summary estimates were pooled with a random effects model and meta-regression explored whether any difference was associated with study-level factors.

**Results:**

Of 21,527 identified studies, 59 were eligible (11,101,581 participants). Crude global hypertension risk was lower among PLHIV than HIV-negative individuals (risk ratio 0.90, 95% CI 0.85–0.96), although heterogeneity between studies was high (*I*^2^ = 97%, *p* < 0.0001). The relationship varied by continent, with risk higher among PLHIV in North America (1.12, 1.02–1.23) and lower among PLHIV in Africa (0.75, 0.68–0.83) and Asia (0.77, 0.63–0.95). Meta-regression revealed strong evidence of a difference in risk ratios when comparing North American and European studies to African ones (North America 1.45, 1.21–1.74; Europe 1.20, 1.03–1.40).

**Conclusions:**

Our findings suggest that the relationship between HIV status and prevalent hypertension differs by region. The results highlight the need to tailor hypertension prevention and care to local contexts and underscore the importance of rapidly optimising integration of services for HIV and hypertension in the worst affected regions. The role of different risk factors for hypertension in driving context-specific trends remains unclear, so development of further cohorts of PLHIV and HIV-negative controls focused on this would also be valuable.

## Introduction

The introduction of antiretroviral therapy (ART) has had a substantial impact on the life expectancy of people living with HIV (PLHIV) [1]. As a result, PLHIV are increasingly ageing and affected by non-communicable diseases (NCDs), including hypertension [2]. Hypertension, though often asymptomatic, is a key risk factor for other NCDs, such as chronic kidney disease and cardiovascular disease [3], and can be controlled or uncontrolled in the presence of antihypertensives.

In recent years, a number of observational studies have found a differing prevalence of hypertension among PLHIV compared to HIV-negative individuals, with some finding an elevated burden [4, 5], and others finding a reduced burden [6, 7]. Possible causes of elevated hypertension prevalence in PLHIV include chronic inflammation, increased microbial translocation and renal disease, blood vessel damage resulting from long-term ART exposure, and higher levels of behavioural risk factors among PLHIV in some communities [8, 9]. Conversely, possible reasons for reduced hypertension burden among PLHIV include low blood pressure resulting from advanced HIV disease, better control of blood pressure due to additional healthcare support and lower levels of behavioural risk factors among PLHIV in some settings [7, 10, 11]. Limited comparability between PLHIV and HIV-negative controls in observational studies may also explain some of the differencing conclusions [12]. Despite the various plausible mechanisms and inconsistent evidence from observational research, no study to date has systematically established whether there is a global difference in hypertension by HIV status, nor its directionality. Such data will be vital to inform policy decisions on optimising hypertension prevention and care.

We aimed to perform a global systematic review and meta-analysis of cross-sectional studies to assess whether there is a difference in risk of prevalent hypertension by HIV status among adults aged over 15. We also aimed to complete a meta-regression to explore whether any differences were associated with specific study-level factors.

## Methods

### Search strategy and selection criteria

We carried out a global systematic review and meta-analysis of cross-sectional studies to establish whether the risk of prevalent hypertension differs by HIV status, following the Preferred Reporting Items for Systematic Reviews and Meta-Analyses (PRISMA) guidelines [13]. We searched MEDLINE, EMBASE, Global Health and Cochrane CENTRAL Register of Controlled Trials from inception to August 23, 2020, using a structured search strategy to identify cross-sectional studies assessing the risk of prevalent hypertension in HIV-positive and HIV-negative individuals aged over 15. Searches were not restricted by language or quality of study, although unpublished reports and reports in conference abstracts were excluded to minimise the effect of selective reporting.

Full details on PRISMA compliance, inclusion criteria, search strategy, data collection and the process for contacting authors are in Additional file 1. Briefly, we included studies that used the following definitions for hypertension:

i) Blood pressure measurement thresholds equivalent to or higher than a systolic blood pressure (SBP) of 130 mmHg or a diastolic blood pressure (DBP) of 80 mmHg [14]
ii) Use of antihypertensive medication
iii) Electronic health record of a hypertension diagnosis

Studies investigating non-systemic hypertension (e.g. intracranial, pulmonary and portal hypertension) or that recruited or rejected people with conditions associated with hypertension (e.g., kidney disease, heart disease or diabetes mellitus) were excluded. In addition, studies of hospitalised populations or focusing solely on key populations (men who have sex with men, transgender people, commercial sex workers, people who inject drugs, prisoners and migrants) were excluded to reduce threats to external validity of the findings for the broader population of PLHIV.

After duplicate and retracted reports were removed, titles and abstracts were independently screened by KD and PPG, with conflicts resolved by MS. Full texts of remaining studies were assessed in the same way. Reference lists of included full-text articles were screened to identify additional studies. If several reports were identified that used the same study population, only the most recent or comprehensive study was included.

Three authors (KD, PPG and MS) independently extracted the data from studies, using a form which had been piloted on ten studies and refined accordingly. Summary estimates, rather than individual-level data, were extracted. Data extracted included first author, publication year, country of recruitment, study period, hypertension definition, sample size, age limits for inclusion, average participant age, gender distribution, percentage of PLHIV on ART and hypertension prevalence information. Data on hypertension prevalence included on number with hypertension and total number of people in each group, as well as reported or calculated standard error. Where relevant data was not available, corresponding authors were contacted.

### Data analysis

Crude risk ratios (RRs) and 95% confidence intervals (CIs) by HIV status were obtained from studies. Unadjusted estimates were used to aid comparability across studies. Where studies did not report RRs, they were calculated by dividing risk of prevalent hypertension among PLHIV by risk of prevalent hypertension among HIV-negative individuals (Eq. 1).

$$ \mathrm{RR}=\frac{\mathrm{Risk}\ \mathrm{of}\ \mathrm{prevalent}\ \mathrm{hypertension}\ \mathrm{in}\ \mathrm{PLHIV}}{\mathrm{Risk}\ \mathrm{of}\ \mathrm{prevalent}\ \mathrm{hypertension}\ \mathrm{in}\ \mathrm{HIV}\ \mathrm{negative}\ \mathrm{in}\mathrm{dividuals}}\#1 $$

95% CIs were then calculated using the R package “meta” [15].

RRs were pooled with random effects models to adjust for variance within and between studies, using the Mantel-Haenszel method [16]. Statistical heterogeneity was assessed using the chi-squared test for heterogeneity on Cochran’s *Q* [17]. Heterogeneity was quantified using the *I*^2^ statistic [18].

Sub-group analyses were performed to assess the impact of continent and hypertension definition on the pooled RR. The role of continent was examined because access to healthcare and characteristics of those at risk of, and living with, hypertension and HIV vary widely by region [19, 20]. Hypertension definition was also examined as the various hypertension definitions may bias the RR in different ways. For example, studies which defined hypertension via electronic health records may detect a relatively higher number of cases of hypertension in PLHIV because PLHIV have more contact with health services, so are more likely to have hypertension diagnoses added to their medical records.

Meta-regression was used to explore the contribution of continent, hypertension definition, mean age, the proportion of PLHIV on ART, the proportion of female participants and the year the study began, to the RR [21]. Variables used in the meta-regression were limited to those for which there was evidence of a plausible clinical or epidemiological effect and that were collected by enough studies to ensure statistical power [22–25]. Study data was examined to ensure the optimal choice of continuous or categorical coding. Several categories of hypertension definitions were combined for statistical power; “SBP≥140mmHg or DBP≥90mmHg or use of antihypertensives”, “SBP≥140mmHg or DBP≥90mmHg” and “Use of antihypertensives” were coded as “Includes SBP≥140mmHg or DBP≥90mmHg or use of antihypertensives”. Studies that defined hypertension using electronic health records were kept as a separate category. All further definitions were coded as “Other”. The year a study began was coded as a categorical variable with two levels, “before 2005” and “2005 onwards”, to reflect the increased availability of effective simplified and more tolerable ART regimens after 2005 [26].

Univariable associations between each variable and the RR were examined. Those for which strong evidence of an effect was found in the univariable analysis (*p* < 0.05 and clinically relevant effect size) were included in a multivariable meta-regression model. A permutation test was used to examine the robustness of meta-regression results [27].

The methodological quality, and comparability of PLHIV and HIV-negative individuals, recruited by included studies was assessed by two reviewers (KD and MS), using the National Heart, Lung and Blood Institute Study Quality Assessment Tools [28]. Any studies considered to be at high risk of bias were excluded from the quantitative analysis in a sensitivity analysis. Publication bias was evaluated by examining symmetry of contour-enhanced funnel plots and using Egger’s test, if there were more than ten studies in the funnel plot [29]. A pre-specified sensitivity analysis was performed, in which the Hartung-Knapp modification was applied to account for small sample sizes [30]. Further sensitivity analyses exploring the effect of using  Joint United Nations Programme on HIV/AIDS ( UNAIDS) regions rather than continents for sub-group analyses were also carried out (Additional file 1).

Analyses were performed in R version 4.0.2, using the packages “meta” and “metafor” [15, 31].

The design and conduct of this review were specified in advance and documented in a protocol. Details were registered on PROSPERO (CRD42019151359, https://www.crd.york.ac.uk/prospero/display_record.php?RecordID=151359).

## Results

### Study selection and characteristics

**Fig. 1:**
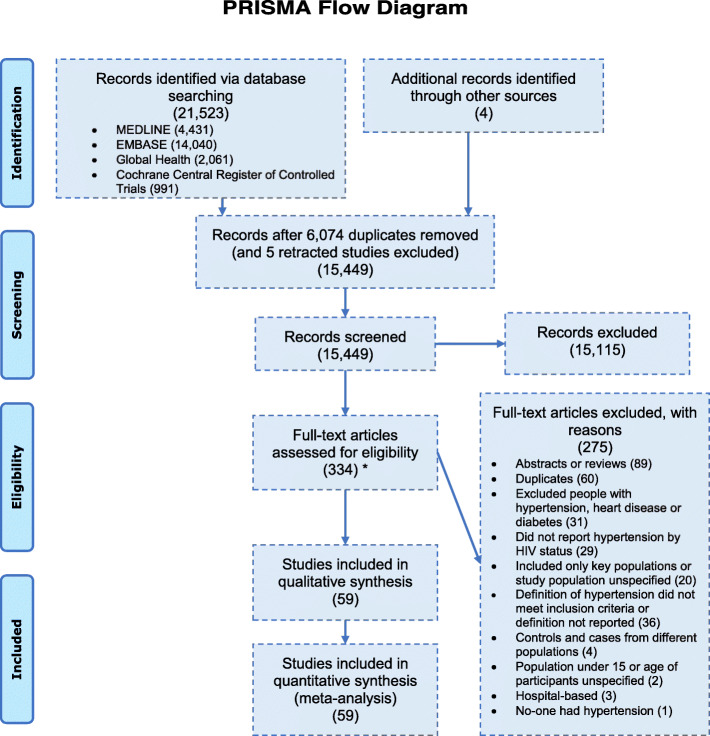
PRISMA flow diagram of the study selection process. The asterick indicates that this is the number of records selected, based on title and abstract

The database searches identified 21,523 records, with three more records identified through reference checking and one study recommended by a study author who was contacted for further information. After 6074 duplicate and retracted reports were removed, 15,449 remained, 334 of which were assessed in a full-text review. Of these, 59 were included in qualitative and quantitative syntheses (Fig. 1 and Additional file 1).

**Fig. 2:**
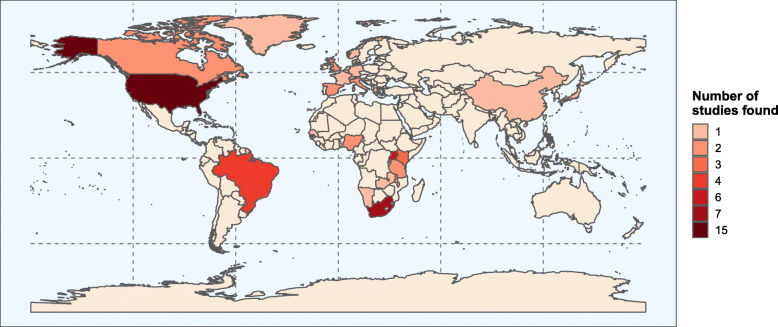
Global distribution of sites used in included studies

The studies had a combined total of 11,101,581 participants, with data collection occurring between 1985 and 2018 (Table 1). Most studies took place in Africa (*n* = 22/59, 37.3%) and North America (*n* = 18/59, 30.5%), with two studies from Asia (3.3%) (Fig. 2). The gender ratio among study participants varied substantially. Mean or median age across many studies fell between 30 and 50 years (*n* = 33/51, 64.7%). Reassuringly, nearly all studies had age-matched controls, meaning that differences in hypertension prevalence are unlikely to be linked to age differences. In most studies, the majority of PLHIV were on ART (*n* = 34/40, 85%), and the most common hypertension definition was “SBP≥140mmHg, DBP≥90mmHg, or use of antihypertensives” (*n* = 23/59, 39.0%).

**Table 1:** Observational studies of hypertension in people living with HIV and HIV-negative individuals that met the inclusion criteria

Authors,year	Continent	Country	Study period	Hypertensiondefinition	Age included(years)	Number ofparticipants	Mean age(years)^**a**^	Proportionfemale (%)	Proportion of people livingwith HIV who were exposedto antiretroviral therapy (%)	Risk ratio forhypertension^**b**^ (95%confidence interval)
Ake et al., 2018 [32]	Africa	Kenya, Uganda, Tanzania and Nigeria	2013–2016	SBP ≥ 140 mmHg or DBP ≥ 90 mmHg or use of antihypertensives	≥ 18	2538	38.5^a^	59	66	0.67 (0.53–0.85)
Akl et al., 2016 [33]	South American	Brazil	Not specified	SBP ≥ 130 mmHg or DBP ≥ 85 mmHg	40–60	388	48.7	100	91	1.03 (0.85–1.26)
Benzekri et al., 2018 [34]	Africa	Senegal	1994–2015	SBP ≥ 140 mmHg or DBP ≥ 90 mmHg	≥ 18	2120	37.2	66.9	14.1	0.61 (0.50–0.75)
Bergersen et al., 2003 [35]	Europe	Norway	2000–2001	SBP ≥ 140 mmHg or DBP ≥ 90 mmHg or use of antihypertensives	Adults^c^	657	43.2	81	100	0.88 (0.65–1.19)
Bonfanti et al., 2007 [36]	Europe	Italy	1995–2005	Use of antihypertensives	≥ 18	3135	47.8	41.3	85.0	0.53 (0.43–0.65)
Burkhalter et al., 2014 [37]	North America	Haiti	2013	SBP ≥ 140 mmHg or DBP ≥ 90 mmHg or use of antihypertensives	≥ 18	608	54.2^a^	64.5	Not specified	1.03 (0.51–2.07)
Chhabra et al., 2018 [38]	Europe	UK	2013–2016	SBP ≥ 140 mmHg or DBP ≥ 90 mmHg or use of antihypertensives	≥ 50	88	Living with HIV 58^a^ HIV-negative 60^a^	17.1	100	1.34 (0.79–2.27)
Chow et al., 2012 [39]	North America	USA	2005–2007	Electronic health records	≥ 18	36,731	40.9	34.2	49	1.19 (1.14–1.24)
Clark et al., 2015 [40]	Africa	South Africa	2014–2015	SBP ≥ 140 mmHg or DBP ≥ 90 mmHg or use of antihypertensives	≥ 15	4350	Not specified	59.4	Not specified	0.92 (0.85–1.00)
Cortes et al., 2017 [41]	North America	USA	2002–2014	Use of antihypertensives	≥ 40	152	57.3	100	78.9	0.86 (0.66–1.11)
Crystal et al., 2011 [42]	North America	USA	2005–2008	SBP ≥ 140 mmHg or DBP ≥ 90 mmHg	Adults^c^	1325	Not specified	100	Not specified	1.13 (1.04–1.22)
Ding et al., 2017 [43]	Asia	China	2014–2015	SBP ≥ 140 mmHg or DBP ≥ 90 mmHg or use of antihypertensives	≥ 40	690	52.8	33.3	87	0.75 (0.58–0.95)
Drain et al., 2019 [23]	Africa	South Africa	2013–2017	SBP ≥ 140 mmHg or DBP ≥ 90 mmHg	≥ 18	5428	31	49.4	0	0.89 (0.75–1.05)
Durand et al., 2011 [44]	North America	Canada	1985–2007	Electronic health records	≥ 20	34,734	39.7	22	76.2	1.44 (1.38–1.52)
Echeverria et al., 2014 [45]	Europe	Spain	2010–2011	SBP ≥ 140 mmHg or DBP ≥ 90 mmHg or use of antihypertensives	≥ 18	254	46.5 ^a^	28.6	94.4	1.03 (0.55–1.94)
Gallant et al., 2017 [46]	North America	USA	2003–2013	Electronic health records	≥ 18	121,738	45.8	29	100	1.06 (1.04–1.08)
Gelpi et al., 2018 [47]	Europe	Denmark	2015–2016	SBP ≥ 140 mmHg or DBP ≥ 90 mmHg or use of antihypertensives	≥ 18	13,260	Living with HIV 50.1^a^ HIV-negative 52.2^a^	18.3	98.4	0.72 (0.67–0.77)
Godijk et al., 2020 [48]	Africa	South Africa	2014–2017	SBP ≥ 140 mmHg or DBP ≥ 90 mmHg	≥ 18	318	41.9	47.3	100	0.93 (0.81–1.08)
Guaraldi et al., 2011 [49]	Europe	Italy	2002–2019	Electronic health records	≥ 18	11,416	46	37	100	1.13 (1.04–1.22)
Guaraldi et al., 2018 [50]	Europe	Italy	2015–2016	SBP ≥ 140 mmHg or DBP ≥ 90 mmHg or use of antihypertensives	≥ 65	1183	71.6	17.2	100	0.95 (0.87–1.04)
Hasse et al., 2015 [51]	Europe	Switzerland	2009–2011	SBP ≥ 160 mmHg or DBP ≥ 90 mmHg or use of antihypertensives	≥ 40	7799	Living with HIV 50^a^ HIV-negative 57^a^	39.2	Not specified	0.96 (0.92–1.01)
Hendriks et al. 2012 [52]	Africa	Kenya, Namibia, Tanzania and Nigeria	2009–2011	SBP ≥ 140 mmHg or DBP ≥ 90 mmHg or use of antihypertensives	≥ 15	1848	41.0	56.6	Not specified	1.06 (0.65–1.71)
Hopkins et al., 2019 [53]	Africa	South Africa	2018	SBP ≥ 140 mmHg or DBP ≥ 90 mmHg	≥ 18	136	Not specified	51.1	Not specified	0.67 (0.37–1.20)
Jerico et al., 2005 [54]	Europe	Spain	2003	SBP ≥ 140 mmHg or DBP ≥ 90 mmHg or use of antihypertensives	≥ 20	1512	42.3	29.6	88	0.97 (0.75–1.26)
Kavishe et al., 2015 [55]	Africa	Tanzania and Uganda	2012–2013	SBP ≥ 140 mmHg or DBP ≥ 90 mmHg or use of antihypertensives	≥ 18	1928	Not specified	56	Not specified	2.00 (1.51–2.66)
Kelly et al., 2008 [56]	Africa	Zambia	2003	SBP ≥ 140 mmHg or DBP ≥ 90 mmHg	≥ 18	312	34.2	67	0	0.40 (0.22–0.72)
Kingery et al., 2016 [57]	Africa	Tanzania	2012–2013	SBP ≥ 140 mmHg or DBP ≥ 90 mmHg	≥ 18	454	40.5	65.6	49.8	1.04 (0.67–1.61)
Klein et al., 2015 [58]	North America	USA	1996–2011	Electronic health records	≥ 18	282,368	50	9	90	0.89 (0.85–0.93)
Kunisaki et al., 2015 [59]	North America	USA	2002–2010	Electronic health records	≥ 18	7324	49.9	4.9	72.7	0.55 (0.52–0.59)
Kwarisiima et al., 2016 [60]	Africa	Uganda	Not specified	SBP ≥ 140 mmHg or DBP ≥ 90 mmHg or use of antihypertensives	≥ 18	59,631	34^a^	59	Not specified	0.79 (0.71–0.86)
Maciel et al., 2018 [61]	South America	Brazil	2016	SBP ≥ 140 mmHg or DBP ≥ 90 mmHg or use of antihypertensives	≥ 50	416	57^a^	44.2	98.1	0.89 (0.77–1.02)
Malaza et al., 2012 [24]	Africa	South Africa	2010	SBP ≥ 140 mmHg or DBP ≥ 90 mmHg	≥ 15	10,429	Not specified	Not specified	Not specified	0.70 (0.64–0.76)
Masyuko et al., 2020 [62]	Africa	Kenya	2017–2018	SBP ≥ 130 mmHg or DBP ≥ 85 mmHg	≥ 30	598	Living with HIV 45^a^ HIV-negative 40^a^	50	100	0.64 (0.49–0.84)
Mayer et al., 2018 [4]	North America	USA	2006–2016	Electronic health records	≥ 18	239,849	41	63.6	Not specified	1.22 (1.19–1.24)
Minami et al., 2019 [63]	Asia	Japan	Not specified	SBP ≥ 140 mmHg or DBP ≥ 90 mmHg or antihypertensives or electronic health records	≥ 18	472	Living with HIV 39^a^ HIV-negative 39.5^a^	4.4	100	0.86 (0.57–1.29)
Mondy et al., 2007 [64]	North America	USA	2001–2005	SBP ≥ 130 mmHg or DBP ≥ 85 mmHg or use of antihypertensives	Adults^c^	942	43.4	34.5	69	0.85 (0.71–1.02)
Monteiro et al., 2012 [65]	South America	Brazil	2009	SBP ≥ 140 mmHg or DBP ≥ 90 mmHg or use of antihypertensives	≥ 18	343	42	46.9	89%	0.63 (0.42–0.94)
Mosha et al., 2017 [6]	Africa	Tanzania	2012–2013	SBP ≥ 140 mmHg or DBP ≥ 90 mmHg	≥ 15	9678	29^a^	64.9	Not specified	0.64 (0.47–0.88)
Nakanga et al., 2019 [66]	Africa	Malawi	2013–2016	SBP ≥ 140 mmHg or DBP ≥ 90 mmHg or use of antihypertensives	≥ 15	3306	Not specified	Not specified	Not specified	0.76 (0.58–0.98)
Nakibuuka et al., 2015 [67]	Africa	Uganda	2012–2013	SBP ≥ 140 mmHg or DBP ≥ 90 mmHg or use of antihypertensives	≥ 18	4967	34.7	69.4	Not specified	0.60 (0.48–0.75)
Odden et al., 2007 [68]	North America	USA	Not specified	SBP ≥ 140 mmHg or DBP ≥ 90 mmHg or use of antihypertensives	33–45	809	39.7	36.1	Not specified	1.43 (1.04–1.95)
Okello et al., 2017 [7]	Africa	Uganda	2015	SBP ≥ 140 mmHg or DBP ≥ 90 mmHg	≥ 40	1115	Living with HIV 45^a^ HIV-negative 46^a^	56.6	Not specified	0.75 (0.61–0.92)
Pacheco et al., 2016 [69]	South America	Brazil	2011–2012	SBP ≥ 140 mmHg or DBP ≥ 90 mmHg or use of antihypertensives	Adults^c^	623	Living with HIV 43.6^a^ HIV-negative 44.5^a^	45.9	89	0.75 (0.57–0.98)
Rucker et al., 2018 [70]	Africa	Malawi	2015–2016	SBP ≥ 140 mmHg or DBP ≥ 90 mmHg	≥ 30	735	Living with HIV 47^a^ HIV-negative 52^a^	73.7	100	0.76 (0.58–0.98)
Russell et al., 2020 [71]	North America	Canada	2013–2017	SBP ≥ 140 mmHg or DBP ≥ 90 mmHg or use of antihypertensives	≥ 15	289	44.1	100	92	1.57 (0.83–2.97)
Ryscavage et al., 2019 [72]	North America	USA	2012–2014	SBP ≥ 140 mmHg or DBP ≥ 90 mmHg or use of antihypertensives	18–29	324	24^a^	58	82.4	2.00 (1.00–4.00)
Sanders et al., 2017 [73]	North America	USA	2000–2016	Electronic health records	≥ 18	15,173	48.2	17.3	Not specified	1.77 (1.67–1.87)
Sarfo et al., 2019 [74]	Africa	Ghana	Not specified	SBP ≥ 140 mmHg or DBP ≥ 90 mmHg	≥ 30	701	44.6	81.2	55.4	0.65 (0.50–0.84)
Saves et al., 2003 [75]	Europe	France	1995–1999	SBP ≥ 160 mmHg or DBP ≥ 95 mmHg	35–44	1312	Not specified	43	100	0.51 (0.29–0.90)
Savinelli et al., 2020 [76]	Europe	UK	2013–2016	Electronic health records	≥ 50	1361	53.4	19.2	100	1.01 (0.79–1.29)
Scholten et al., 2011 [77]	Africa	Uganda	2009–2010	SBP ≥ 140 mmHg or DBP ≥ 90 mmHg	≥ 50	511	65.8	61.2	50.8	0.47 (0.34–0.65)
Schutte et al., 2012 [78]	Africa	South Africa	2005–2010	Use of antihypertensives	≥ 30	332	46	65.3	Not specified	0.49 (0.22–1.06)
Touloumi et al., 2020 [79]	Europe	Greece	1996–2014	SBP ≥ 140 mmHg or DBP ≥ 90 mmHg or use of antihypertensives	≥ 18	10,659	41.6^a^	13.1	86.1	1.33 (1.15–1.53)
Triant et al., 2007 [80]	North America	USA	1996–2004	Electronic health records	18–84	1,048,440	Living with HIV 38^a^ HIV-negative 39^a^	59	41	1.34 (1.26–1.42)
van Heerden et al., 2017 [81]	Africa	South Africa	2015	SBP ≥ 140 mmHg or DBP ≥ 90 mmHg	≥ 18	570	Not specified	69	Not specified	0.64 (0.49–0.85)
van Zoest et al., 2016 [5]	Europe	Netherlands	2010–2012	SBP ≥ 140 mmHg or DBP ≥ 90 mmHg or use of antihypertensives	≥ 45	1044	Living with HIV 52.2^a^ HIV-negative 52.9^a^	12.9	94.7	1.33 (1.15–1.53)
Watson et al., 2017 [82]	North America	USA	Not specified	SBP ≥ 140 mmHg or DBP ≥ 90 mmHg	≥ 60	90	Living with HIV 63^a^ HIV-negative 65^a^	8.9	97	1.29 (0.84–1.97)
Yang et al., 2019 [83]	North America	USA	2007–2016	Electronic health records	≥ 50	9,141,867	64.1	Not specified	Not specified	1.28 (0.94–1.74)
Yu et al., 2019 [84]	North America	USA	2013–2016	SBP ≥ 130 mmHg or DBP ≥ 85 mmHg	35–65	201	50.8	22.9	95.4	1.28 (0.94–1.74)

*SBP* systolic blood pressure, *DBP* diastolic blood pressure

^a^Median ages are reported where mean values were not available

^b^A risk ratio greater than one indicates that the prevalence is higher in PLHIV, and a risk ratio less than one indicates that the prevalence is lower in PLHIV

^c^Studies in Europe, North and South America which reported that they had included only adults were assumed to have only included participants over the age of 14

### Synthesis and sensitivity analyses

**Fig. 3:**
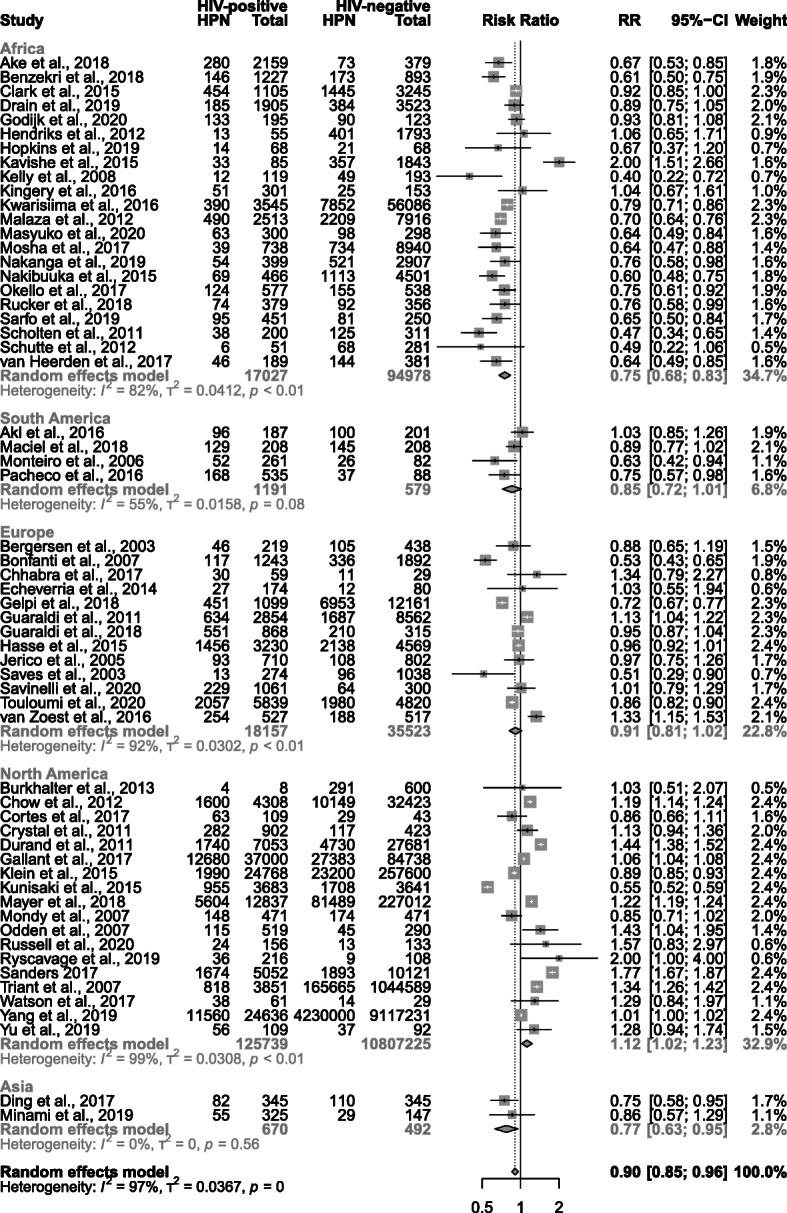
Forest plot of the risk ratio for hypertension by HIV status, categorised by continent. Values less than one indicate a lower hypertension prevalence in people living with HIV. Estimates were pooled using a random effects model, with a *p*-value for sub-group differences less than 0.0001. RR risk ratio, CI confidence interval

The overall risk of crude prevalent hypertension was lower among PLHIV than HIV-negative individuals (RR = 0.90, 95% CI 0.85–0.96) (Fig. 3), although there was strong evidence of heterogeneity between studies (*I*^2^ 97%, *p* < 0.0001).

Analysis by continent revealed considerable differences (*p* < 0.0001), with a RR of 0.75 (95% CI 0.68–0.83) for African studies and a RR of 0.77 (95% CI 0.63–0.95) for Asian studies contrasting with a RR of 1.12 (95% CI 1.02–1.23) for North American studies. We did not find evidence that the RR differed among European (RR = 0.91, 95% CI 0.81–1.02) or South American studies (RR = 0.85, 0.72–1.01). Analysis also highlighted heterogeneity associated with the definition of hypertension (*p* = 0.0001, Additional file 1). Studies defining hypertension as “SBP≥140mmHg or DBP≥90mmHg” (RR = 0.75, 0.66–0.85), “Use of antihypertensives” (RR = 0.63, 0.41–0.96) or “SBP≥160mmHg or DBP≥95mmHg” (RR = 0.51, 0.29–0.90) reported lower prevalence among PLHIV. By contrast, strong evidence for a difference was not established for any other hypertension definition sub-group.

In univariate meta-regression analyses, only continent and hypertension definition were predictors of the RR (Additional file 1). Multivariable meta-regression including these two predictors revealed that the effect of hypertension definition was lost after controlling for continent (Table 2). The meta-regression results provided strong evidence that the RR for hypertension by HIV status was higher in North America and Europe than Africa (North America RR = 1.45, 95% CI 1.21–1.74; Europe 1.20, 1.03–1.40). We did not find evidence of a difference when comparing Africa to the other continents (Table 2). A permutation test revealed results that were largely in line with the original model, suggesting that these findings are robust (Additional file 1).

**Table 2:** Multivariable meta-regression (*R*^2^ = 13.88%)

	Number of studies	Number of participants	Risk ratio (95% confidence interval)	***p***-value for sub-category	Overall ***p***-value
Hypertension definition					**0.002**
Includes systolic blood pressure ≥ 140 mmHg or diastolic blood pressure ≥ 90 mmHg and/or use of antihypertensives	41	148,868	1	–	
Electronic health records	11	10,941,001	1.04 (0.87–1.24)	0.643	
Other	7	11,712	0.97 (0.80–1.17)	0.748	
Continent					0.657
Africa	22	112,005	1	–	
Asia	2	1162	1.06 (0.74–1.50)	0.763	
Europe	13	53,680	1.20 (1.03–1.40)	**0.019**	
North America	18	10,932,964	1.45 (1.21–1.74)	**< 0.001**	
South America	4	1770	1.13 (0.89–1.43)	0.327	

Two studies were found to be at high risk of bias. They did not recruit all their subjects from similar populations, justify their sample size or apply inclusion and exclusion criteria uniformly (Additional file 1) [34, 36]. The remaining studies were of low or medium risk of bias. Removing the two studies at high risk of bias did not substantially alter our results (Additional file 1).

Visual inspection of a contour-enhanced funnel plot including all studies, and the use of Egger’s test, revealed strong evidence of asymmetry (*p* = 0.049). While this may indicate publication bias, contour-enhanced funnel plots and Egger’s tests for each continent did not reveal substantial asymmetry, suggesting the asymmetry in the overall plot is due to RR variation by location (Additional file 1). Sensitivity analyses using the Hartung-Knapp modification widened the confidence intervals around the overall pooled RR, although the evidence of a difference remained clear (RR = 0.90, 0.83–0.98). Analysis by the UNAIDS region revealed results which corroborated findings by continent. For example, hypertension risk was lower among PLHIV for studies in East and Southern Africa (RR = 0.76, 0.68–0.86), West and Central Africa (0.63, 0.54–0.74) and Asia and the Pacific (0.77, 0.63–0.95). Full details on sensitivity analyses are in Additional file 1.

## Discussion

### Summary of evidence

To our knowledge, this is the first global systematic review and meta-analysis to compare the risk of prevalent hypertension among PLHIV and HIV-negative individuals. We found that the overall global crude risk of prevalent hypertension was lower among PLHIV than HIV-negative individuals. However, there was a high degree of heterogeneity between studies. Sub-group analyses revealed that the relationship varied greatly by region, with risk higher among PLHIV in North America and lower among PLHIV in Africa and Asia. Meta-regression confirmed this, providing strong evidence of a difference in the pooled RR between North American studies and African studies, and between European studies and African studies.

### Evidence in context

Our findings are in line with previous studies, which have contrasted global estimates without ensuring comparability between populations. A 2017 systematic review estimating the global prevalence of hypertension among PLHIV reported that approximately 25% of PLHIV had hypertension; when the authors compared their prevalence with a separate global prevalence estimate for the general population, they found that their result from PLHIV was lower [85]. A second recent review on the same topic found a similar prevalence estimate for PLHIV [86]. Our results by continent are also consistent with regional systematic review data [10, 86, 87]. For example, Dillon et al. performed a systematic review of blood pressure by HIV status in sub-Saharan Africa and found that HIV infection was associated with lower DBP and SBP [10]. Similarly, Bigna et al. carried out a systematic review on risk of prevalent hypertension, which focused only on PLHIV, and reported a higher risk of prevalent hypertension in North America and Western and Central Europe compared with other regions [86]. Unlike these existing studies, our analysis was global and only included studies that directly compared the risk of prevalent hypertension between PLHIV and HIV-negative individuals living in the same communities.

Our results also have important policy implications for the management of hypertension and HIV. They highlight the need for health systems globally to provide effective prevention and care for hypertension, to mitigate downstream health impacts including cardiovascular and renal complications [3]. Yet, several studies have demonstrated important gaps in the treatment cascade, with a large proportion of patients either undiagnosed, untreated or with uncontrolled hypertension [88, 89].

Various strategies could be employed by clinicians and policymakers to overcome these challenges for both PLHIV and HIV-negative individuals, with our results suggesting that a tailored, regional approach is required. In areas where the risk of prevalent hypertension is higher among PLHIV, optimising integration of hypertension services into HIV care by capitalising on pre-existing systems and multidisciplinary approaches will be key [90]. Where the risk of prevalent hypertension is lower in PLHIV, it will be essential to consider trade-offs between delivering HIV-specific integrated interventions and population-level approaches for hypertension. Limited resources for integration need to be focused on the diseases with both the largest overlapping burdens with HIV and the most cost-effective strategies to diagnose and treat this dual burden, to ensure the greatest benefit for patients and health systems [90]. It is also important to recognise that geographic patterns in the relationship between HIV and hypertension may shift over time as patterns of ART exposure, severe disease in PLHIV and access to healthcare change; further research focused on trends over time will provide additional insight for policymakers into the extent of these alterations and how they can be managed. Whatever the specific strategies and policies employed, our results highlight the need for locally focused, resilient and adaptable healthcare to respond to the varying challenges posed by hypertension and HIV.

Key to tailored care will be a better understanding of why the burden of hypertension by HIV status may differ by region. One possible hypothesis is that PLHIV in Africa and Asia are more likely to experience advanced HIV disease leading to lower blood pressure [7]. Differences between regions in patterns of previous experiences of ART before the study, which would not have been captured by our measure of ART usage during a study, may also play a role [25]. In addition, patterns of behavioural risk factors and experience of hypertension care by HIV status in a region may influence hypertension prevalence [10, 11]. For example, PLHIV on ART may be more likely to have received healthcare services for hypertension as a result of regular visits to clinics [91]. To clarify the health trajectory and mechanisms involved in NCD development among ageing PLHIV, there has been a push to set-up cohorts of PLHIV and appropriate HIV-negative controls, such as the American Veterans Ageing Cohort Study and Dutch AGE_H_IV Cohort [12]. Our study highlights the importance of these cohorts and the need for additional African and Asian cohorts, which could further clarify setting-specific risk factors by HIV status. Pre-existing cohorts in these settings, such as those run by the Network for Analysing Longitudinal Population-based HIV/AIDS data on Africa (ALPHA network), the African Non-Communicable Disease Longitudinal data Alliance (ANDLA) and Health and Aging in Africa: A Longitudinal Study of an INDEPTH Community in South Africa (HAALSI) can offer insights into the relationships between HIV and hypertension [92, 93]. However, focused, specific cohort studies will ensure that highly comparable controls are recruited and detailed relevant data, including information on diet, exercise and inflammatory markers are gathered. This will allow mechanisms which may underly regional differences in hypertension prevalence to be more precisely identified.

### Strengths and limitations

Our study has several strengths. It is the first global systematic review and meta-analysis to compare the risk of prevalent hypertension among PLHIV and HIV-negative individuals, revealing new insights that may influence hypertension care in coming years. By complementing the systematic review and meta-analysis with in-depth meta-regression and sensitivity analyses, the study was able to determine factors associated with differences in hypertension by HIV status. Many included studies focused on selecting comparable HIV-negative controls, providing robust RR estimates.

However, this review also has some limitations. First, there is heterogeneity in both the populations investigated and the hypertension measurement procedures across the studies. Second, the quality of included studies varied substantially, although only two were found to be at high risk of bias. Third, there was large heterogeneity in the type of additional data collected by studies and the number of studies from each region, which meant that we were unable to examine all factors associated with hypertension, such as body mass index and physical activity levels, in our meta-regression or to fully explore the relationship in Asia [94]. HIV-related information, including patterns of exposure to ART prior to the study, viral suppression statistics and CD4 counts, was also rare. The lack of data makes it hard to pinpoint why the differences we report in hypertension prevalence by HIV status occurred. Moreover, few included studies (approximately 12) provided any adjusted estimates for risk of prevalent hypertension by HIV status; this was mainly because the focus of the paper was on a different, but related objective. When studies did provide adjusted estimates, the variables which were selected for adjustment differed considerably. It was, therefore, not possible to perform a comprehensive analysis of adjusted estimates. Further studies with a more systematic approach to collecting data on hypertension risk factors will allow more detailed analysis.

## Conclusions

This study suggests that the relationship between HIV status and risk of prevalent hypertension differs by region. The results emphasise the need for hypertension prevention and care to be tailored to the local epidemiological context and for further cohorts of PLHIV and HIV-negative controls to ascertain mechanisms driving these context-specific trends.

## Supplementary Information

**Additional file 1.** Supplementary material. Combined supporting information. This file contains the PRISMA checklist, further details on the search process and additional results.

## Data Availability

All data generated or analysed during this study are included in this published article and its supplementary information files.

## Abbreviations

ANDLA: African Non-Communicable Disease Longitudinal data Alliance
ALPHA: Analysing Longitudinal Population-based HIV/AIDS data on Africa
ART: Antiretroviral therapy
CI: Confidence interval
DBP: Diastolic blood pressure
HAALSI: Health and Aging in Africa: A Longitudinal Study of an INDE PTH Community in South Africa
NCD: Non-communicable disease
PRISMA: Preferred Reporting Items for Systematic Reviews and Meta-Analyses
PLHIV: People living with HIV
RR: Risk ratio
SBP: Systolic blood pressure
UNAIDS: Joint United Nations Programme on HIV/AIDS
