# Correction to: Association between HIV infection and hypertension: a global systematic review and meta-analysis of cross-sectional studies

**DOI:** 10.1186/s12916-021-02112-3

**Published:** 2021-09-08

**Authors:** Katherine Davis, Pablo Perez-Guzman, Annika Hoyer, Ralph Brinks, Edward Gregg, Keri N. Althoff, Amy C. Justice, Peter Reiss, Simon Gregson, Mikaela Smit

**Affiliations:** 1 MRC Centre for Global Infectious Disease Analysis, Department of Infectious Disease Epidemiology, St Mary’s Campus, Imperial College London, London, W2 1PG UK; 2 Department of Statistics, Ludwig-Maximilians-University Munich, Munich, Germany; 3 Hiller Research Unit of Rheumatology, University Hospital Duesseldorf, Duesseldorf, Germany; 4 Department of Epidemiology and Biostatistics, Imperial College London, London, UK; 5 Department of Epidemiology, Johns Hopkins University, Baltimore, MD USA; 6 Schools of Medicine and Public Health, Yale University, New Haven, CT USA; 7 Department of Global Health, Amsterdam University Medical Centers, University of Amsterdam and Amsterdam Institute for Global Health and Development, Amsterdam, Netherlands; 8 HIV Monitoring Foundation, Amsterdam, Netherlands; 9 Biomedical Research and Training Institute, Harare, Zimbabwe

**Correction to: BMC Med 19, 105 (2021)**

**https://doi.org/10.1186/s12916-021-01978-7**

The original article [1] contains an error in Table 1 whereby it is stated that the study period of the observational study by Guaraldi et al. was 2002–2019.

This should instead state 2002–**2009**.
