# Supplementary material for: Association between HIV infection and hypertension: a global systematic review and meta-analysis of cross-sectional studies
Source: BMC Med. 2021 May 13;19:105. doi: 10.1186/s12916-021-01978-7 (PMC8117497; doi:10.1186/s12916-021-01978-7)
Supplement: Supplementary file 1 — Additional file 1. Supplementary material. Combined supporting information. This file contains the PRISMA checklist, further details on the search process and additional results. [file 12916_2021_1978_MOESM1_ESM.pdf]

Supplementary material

PRISMA checklist 2
Additional inclusion criteria details 4
Search strategy 5
Data extraction sheet 8
Method for contacting authors 9
References for included studies 11
Forest plot showing risk ratios by hypertension definition 15
Univariate meta-regression results 16
Permutation test results 17
Risk of bias assessment 18
Results with high-risk studies removed 21
Contour-enhanced funnel plots and Egger's test results 23
Results with Hartung-Knapp modification 27
UNAIDS regions sensitivity analysis 29

PRISMA checklist

Section/topic	#	Checklist item	Reported on page #
**TITLE**			
Title	1	Identify the report as a systematic review, meta-analysis, or both.	1 - Title
**ABSTRACT**			
Structured summary	2	Provide a structured summary including, as applicable: background; objectives; data sources; study eligibility criteria, participants, and interventions; study appraisal and synthesis methods; results; limitations; conclusions and implications of key findings; systematic review registration number.	2 – Abstract, paragraphs 1-4
**INTRODUCTION**			
Rationale	3	Describe the rationale for the review in the context of what is already known.	3 – Introduction, paragraphs 1 and 2
Objectives	4	Provide an explicit statement of questions being addressed with reference to participants, interventions, comparisons, outcomes, and study design (PICOS).	3 – Introduction, paragraph 3
**METHODS**			
Protocol and registration	5	Indicate if a review protocol exists, if and where it can be accessed (e.g., Web address), and, if available, provide registration information including registration number.	6 – Methods, paragraph 13
Eligibility criteria	6	Specify study characteristics (e.g., PICOS, length of follow-up) and report characteristics (e.g., years considered, language, publication status) used as criteria for eligibility, giving rationale.	3,4 – Methods, paragraphs 1-3
Information sources	7	Describe all information sources (e.g., databases with dates of coverage, contact with study authors to identify additional studies) in the search and date last searched.	3 – Methods, paragraph 1
Search	8	Present full electronic search strategy for at least one database, including any limits used, such that it could be repeated.	Supplementary material
Study selection	9	State the process for selecting studies (i.e., screening, eligibility, included in systematic review, and, if applicable, included in the meta-analysis).	4 - Methods, paragraphs 2-4
Data collection process	10	Describe method of data extraction from reports (e.g., piloted forms, independently, in duplicate) and any processes for obtaining and confirming data from investigators.	4 – Methods, paragraph 5
Data items	11	List and define all variables for which data were sought (e.g., PICOS, funding sources) and any assumptions and simplifications made.	4 – Methods, paragraph 5
Risk of bias in individual studies	12	Describe methods used for assessing risk of bias of individual studies (including specification of whether this was done at the study or outcome level), and how this information is to be used in any data synthesis.	6 – Methods, paragraph 11

Summary measures	13	State the principal summary measures (e.g., risk ratio, difference in means).	5 – Methods, paragraph 6
Synthesis of results	14	Describe the methods of handling data and combining results of studies, if done, including measures of consistency (e.g., I^2) for each meta-analysis.	5- Methods, paragraph 7
Risk of bias across studies	15	Specify any assessment of risk of bias that may affect the cumulative evidence (e.g., publication bias, selective reporting within studies).	6 - Methods, paragraph 11
Additional analyses	16	Describe methods of additional analyses (e.g., sensitivity or subgroup analyses, meta-regression), if done, indicating which were pre-specified.	5,6 -Methods, paragraphs 8-11
**RESULTS**			
Study selection	17	Give numbers of studies screened, assessed for eligibility, and included in the review, with reasons for exclusions at each stage, ideally with a flow diagram.	6 – Results, paragraph 1, Fig. 1
Study characteristics	18	For each study, present characteristics for which data were extracted (e.g., study size, PICOS, follow-up period) and provide the citations.	6-7 – Results paragraph 2, Table 1, Fig. 2
Risk of bias within studies	19	Present data on risk of bias of each study and, if available, any outcome level assessment (see item 12).	8 – Results, paragraph 6
Results of individual studies	20	For all outcomes considered (benefits or harms), present, for each study: (a) simple summary data for each intervention group (b) effect estimates and confidence intervals, ideally with a forest plot.	Table 1, Fig. 3
Synthesis of results	21	Present the main results of the review. If meta-analyses are done, include for each, confidence intervals and measures of consistency	7 – Results, paragraph 3
Risk of bias across studies	22	Present results of any assessment of risk of bias across studies (see Item 15).	8 – Results, paragraph 7
Additional analysis	23	Give results of additional analyses, if done (e.g., sensitivity or subgroup analyses, meta-regression [see Item 16]).	7-8 – Results, paragraphs 4-7, Table 2
**DISCUSSION**			
Summary of evidence	24	Summarize the main findings including the strength of evidence for each main outcome; consider their relevance to key groups (e.g., healthcare providers, users, and policy makers).	9-10 – Discussion, paragraphs 1-6
Limitations	25	Discuss limitations at study and outcome level (e.g., risk of bias), and at review-level (e.g., incomplete retrieval of identified research, reporting bias).	11 – Discussion, paragraph 7
Conclusions	26	Provide a general interpretation of the results in the context of other evidence, and implications for future research.	11 – Discussion, paragraph 8
**FUNDING**			
Funding	27	Describe sources of funding for the systematic review and other support (e.g., supply of data); role of funders for the systematic review.	12 – Funding

Additional inclusion criteria details

Age

Some studies from Europe, North America and South America were not explicit about the age of their participants but instead reported including only “adults” without stating their age. We felt this was sufficient for inclusion, as participants were highly likely to be older than 15.

Study types

The outcome variable was a ratio of prevalence figures at a particular time point, so we included any study reporting on cross-sectional analyses. However, our inclusion criteria allowed cross-sectional studies which were completed as part of larger cohort or case-control studies. For example, cross-sectional studies of participants in the AGEHIV Cohort Study, the Veterans Ageing Cohort Study (VACS) and Health and Aging in Africa: A Longitudinal Study of an INDEPTH Community in South Africa (HAALSI) were used in our analysis.

Search strategy

MEDLINE

1. exp Hypertension/

2. hypertens*.mp.

3. exp Blood Pressure/

4. (blood pressure or bloodpressure).mp. [mp=title, abstract, original title, name of substance word, subject heading word, floating sub-heading word, keyword heading word, organism supplementary concept word, protocol supplementary concept word, rare disease supplementary concept word, unique identifier, synonym

5. 1 or 2 or 3 or 4

6. exp HIV/

7. HIV Infections/

8. Acquired Immunodeficiency Syndrome/

9. (hiv or hiv1 or hiv-1* or hiv2 or hiv-2* or aids or human immuno?deficiency virus or human immune?deficiency virus or acquired immuno?deficiency syndrome or acquired immune?deficiency syndrome or hiv?positive or hiv?infected or people living with hiv or plwh or hiv?seropositiv* or hiv?negative or hiv?uninfected or hiv?seronegativ*).mp. [mp=title, abstract, original title, name of substance word, subject heading word, floating sub-heading word, keyword heading word, organism supplementary concept word, protocol supplementary concept word, rare disease supplementary concept word, unique identifier, synonyms]

10. (human immun* and deficiency virus).mp.

11. (acquired immun* and deficiency syndrome).mp.

12. HIV Seropositivity/

13. HIV Seronegativity/

14. 6 or 7 or 8 or 9 or 10 or 11 or 12 or 13

15. 5 and 14

EMBASE

1. exp hypertension/

2. hypertens*.mp.

3. exp blood pressure/

4. (blood pressure or bloodpressure).mp.

5. 1 or 2 or 3 or 4

6. exp Human immunodeficiency virus/

7. Human immunodeficiency virus infection/

8. acquired immune deficiency syndrome/

9. Human immunodeficiency virus infected patient/

10. (hiv or hiv1 or hiv-1* or hiv2 or hiv-2* or aids or human immuno?deficiency virus or human immune?deficiency virus or acquired immune?deficiency syndrome or acquired immuno?deficiency syndrome or hiv?positive or hiv?infect* or people living with HIV or plwh or hiv?seropositiv* or hiv?negative or hiv?uninfect* or hiv?seronegativ*).mp.

11. (human immun* and deficiency virus).mp.

12. (acquired immun* and deficiency syndrome).mp.

13. 6 or 7 or 8 or 9 or 10 or 11 or 12

14. 5 and 13

Global Health

1. exp hypertension/

2. hypertens*.mp.

3. exp blood pressure/

4. (blood pressure or bloodpressure).mp. [mp=title, abstract, original title, name of substance word, subject heading word, floating sub-heading word, keyword heading word, organism supplementary concept word, protocol supplementary concept word, rare disease supplementary concept word, unique identifier, synonyms]

5. 1 or 2 or 3 or 4

6. exp human immunodeficiency viruses/

7. HIV infections/

8. acquired immune deficiency syndrome/

9. (hiv or hiv1 or hiv-1* or hiv2 or hiv-2* or aids or human immuno?deficiency virus or human immune?deficiency virus or acquired immune?deficiency syndrome or acquired immuno?deficiency syndrome or hiv?positive or hiv?infect* or people living with hiv or plwh or hiv?seropositiv* or hiv?negative or hiv?uninfect* or hiv?seronegativ*).mp. [mp=title, abstract, original title, name of substance word, subject heading word, floating sub-heading word, keyword heading word, organism supplementary concept word, protocol supplementary concept word, rare disease supplementary concept word, unique identifier, synonyms]

10. (human immun* and deficiency virus).mp.

11. (acquired immun* and deficiency syndrome).mp.

12. people living with HIV/

13. 6 or 7 or 8 or 9 or 10 or 11 or 12

14. 5 and 13

Cochrane Central Register of Controlled Trials

1. MeSH descriptor: [Hypertension] explode all trees

2. hypertens*

3. MeSH descriptor: [Blood Pressure] explode all trees

4. blood pressure

5. bloodpressure

6. 1 or 2 or 3 or 4 or 5

7. MeSH descriptor: [HIV] explode all trees

8. MeSH descriptor: [Acquired Immunodeficiency Syndrome] this term only

9. MeSH descriptor: [HIV Infections] this term only

10. MeSH descriptor: [HIV Seroprevalence] explode all trees

11. hiv or hiv2 or hiv-2 or hiv1 or hiv-1 or aids or human immuno?deficiency virus or human immune?deficiency virus or acquired immune?deficiency syndrome or acquired immuno?deficiency syndrome or hiv?positive or hiv?infect* or people living with hiv or plwh or hiv?seropositiv* or hiv?negative or negative or hiv?uninfect* or uninfect* or hiv? seronegtiv* or seronegativ*

12. human immun* and deficiency virus

13. acquired immun* and deficiency syndrome

14. 7 or 8 or 9 or 10 or 11 or 12 or 13

15. 6 and 14

Data extraction sheet

Author, year	Country	Continent	Study start	Study end	Hypertension definition	Age range	Mean age	Median age	Proportion female	Proportion PLHIV on ART	Number of PLHIV	Number of PLHIV with hypertension	Number of HIV-negative people	Number of HIV-negative people with hypertension

PLHIV: People Living with HIV. ART: Anti-retroviral therapy.

Method for contacting authors

Authors of 73 studies were contacted requesting missing data. Replies with requested data were received from 30 authors.

Initial email

Dear << Name >>,

I am contacting you about a **global systematic review and meta-analysis of the prevalence of hypertension among HIV-positive and HIV-negative adults** (PROSPERO ID: CRD42019151359, outline).

With my colleagues, Dr Mikaela Smit, Dr Pablo Perez and Professor Edward Gregg, (in cc), I am part of a multi-disciplinary team based at Imperial College London, which focuses on multi-morbidity in People Living with HIV (PLHIV).

I am writing to request some further information from you about your << Year >> study entitled “<< Title >>”. We believe this study may need to be included in our meta-analysis. Could you please assist us by providing answers to the questions below?

<< Questions >>

We would be very grateful if you could provide us with this information by **<<**Date**>>**. If you need more time to retrieve this information, please let us know an estimated date before <<Date>>. If we do not hear back from you, your study will be excluded from our analysis.

Please don’t hesitate to contact me should you have any queries.

All the best,
Katherine Davis

Questions asked

- What was the age range of your participants and did you have any age limits during recruitment?
- How was hypertension defined? Was this based on blood pressure measurement (if so, what cut-off was used?), self-reported diagnosis or clinical factors (such as use of antihypertensives or medical record review)?
- How many PLHIV (aged over 15) had hypertension? How many did not? How many HIV-negative individuals had hypertension? How many did not?
- Could you describe how the American subjects were recruited? Were they taken from the general population, primary care settings, secondary or tertiary care settings or from medical insurance data? Can you briefly describe these settings?
- Could you provide us with data from your study, but excluding commercial sex workers? Specifically, we are interested in knowing how many PLHIV (aged over 15) had hypertension and how many did not, as well as how many HIV-negative individuals had hypertension and how many did not. We also need to know the mean age of study subjects and proportion that were female, in addition to the proportion of PLHIV on ART, for the study to be included.
- Excluding individuals self-reporting hypertension, how many PLHIV (aged over 15) had hypertension? How many did not? How many HIV-negative individuals had hypertension? How many did not?
- How were HIV-negative individuals recruited? How did your recruitment process ensure that PLHIV came from a similar background to HIV-negative individuals?
- Were your participants recruited from key populations (transwomen, commercial sex workers, prisoners, people who inject drugs and migrants), rather than the general population of PLHIV?

- How did you define weighted vs unweighted visits? Is it the case that unweighted visits relate to the actual number of individual patients by arm? What was the number of patients?

Follow-up email to non-responders

Dear << Name >>,

I hope you are well.

I am writing to ask if you have been able to review our questions about your << Year >> study ("<< Title >>")? Our deadline for responses is **<<Date>>** so we would be grateful if you could look over the questions and respond before then.

Full details of our questions and the systematic review are copied below.

Your replies are key to making our systematic review a success. We appreciate the time and effort that you put into reading this email and answering our questions, and look forward to hearing from you.

All the best,
Katherine

References for included studies

1. Ake JA, Polyak CS, Crowell TA, et al. Noninfectious Comorbidity in the African Cohort Study (AFRICOS). Clin Infect Dis **2018**; 69:639–647.

2. Akl LD, Valadares ALR, Gomes DC, Pinto-Neto AM, Costa-Paiva L. Factors associated with metabolic syndrome in middle-aged women with and without HIV. Menopause **2016**; 22:1398–1399.

3. Benzekri NA, Seydi M, N Doye I, et al. Increasing prevalence of hypertension among HIV-positive and negative adults in Senegal, West Africa, 1994-2015. PLoS One **2018**; 13:e0208635.

4. Bergersen BM, Sandvik L, Dunlop O, Birkeland K, Bruun JN. Prevalence of hypertension in HIV-positive patients on highly active retroviral therapy (HAART) compared with HAART-Naive and HIV-negative controls: results from a Norwegian study of 721 patients. Eur J Clin Microbiol Infect Dis **2003**; 22:731–736.

5. Bonfanti P, Giannattasio C, Ricci E, et al. HIV and metabolic syndrome: A comparison with the general population. J Acquir Immune Defic Syndr **2007**; 45:426–431.

6. Burkhalter F, Sannon H, Mayr M, Dickenmann M, Ernst S. Prevalence and risk factors for chronic kidney disease in a rural region of Haiti. Swiss Med Wkly **2014**; 144:w14067.

7. Chhabra S, Underwood J, Cole JH, et al. Clinical research cerebral MRI findings in HIV-positive subjects and appropriate controls. AIDS **2018**; 32:2077–2081.

8. Chow FC, Regan S, Feske S, Meigs JB, Grinspoon SK, Triant VA. Comparison of ischemic stroke incidence in HIV-infected and non-HIV-infected patients in a US health care system. J Acquir Immune Defic Syndr **2012**; 60:351–358.

9. Clark SJ, Gomez-Olive FX, Houle B, et al. Cardiometabolic disease risk and HIV status in rural South Africa: establishing a baseline. BMC Public Health **2015**; 15:135.

10. Cortes YI, Reame N, Zeana C, Jia H, Ferris DC, Shane E. Cardiovascular Risk in HIV-Infected and Uninfected Postmenopausal Minority Women: Use of the Framingham Risk Score. J Women's Heal **2017**; 26:241–248.

11. Crystal HA, Weedon J, Holman S, et al. Associations of cardiovascular variables and HAART with cognition in middle-aged HIV-infected and uninfected women. J Neurovirol **2011**; 17:469–476.

12. Ding Y, Lin H, Shen W, Wu Q, Gao M, He N. Interaction Effects between HIV and Aging on Selective Neurocognitive Impairment. J Neuroimmune Pharmacol **2017**; 12:661–669.

13. Drain PK, Hong T, Hajat A, et al. Integrating hypertension screening at the time of voluntary HIV testing among adults in South Africa. PLoS One **2019**; 14:e0210161.

14. Durand M, Sheehy O, Baril JG, Lelorier J, Tremblay CL. Association between HIV infection, antiretroviral therapy, and risk of acute myocardial infarction: A cohort and nested case-control study using Québec's Public Health Insurance database. J Acquir Immune Defic Syndr **2011**; 57:245–253.

15. Echeverria P, Bonjoch A, Molto J, et al. Pulse wave velocity as index of arterial stiffness in HIV-infected patients compared with a healthy population. J Acquir Immune Defic Syndr **2014**; 65:50–56.

16. Gallant J, Hsue PY, Shreay S, Meyer N. Comorbidities among US patients with prevalent HIV infection - a trend analysis. J Infect Dis **2017**; 216:1525–1533.

17. Gelpi M, Afzal S, Lundgren J, et al. Higher risk of abdominal obesity, elevated low-density lipoprotein cholesterol, and hypertriglyceridemia, but not of hypertension, in people living with human immunodeficiency virus (HIV): results from the Copenhagen Comorbidity in HIV Infection Study. Clin Infect Dis **2018**; 67:579–586.

18. Godijk NG, Vos AG, Jongen VW, et al. Heart Rate Variability, HIV and the Risk of Cardiovascular Diseases in Rural South Africa. Glob Heart **2020**; 15:17.

19. Guaraldi G, Orlando G, Zona S, et al. Premature age-related comorbidities among HIV-infected persons compared with the general population. Clin Infect Dis **2011**; 53:1120–1126.

20. Guaraldi G, Malagoli A, Calcagno A, et al. The increasing burden and complexity of multi-morbidity

and polypharmacy in geriatric HIV patients: a cross sectional study of people aged 65 - 74 years and more than 75 years. BMC Geriatr **2018**; 18:99.

21. Hasse B, Tarr PE, Marques-Vidal P, et al. Strong Impact of Smoking on Multimorbidity and Cardiovascular Risk Among Human Immunodeficiency Virus-Infected Individuals in Comparison With the General Population. Open Forum Infect Dis **2015**; 2:ofv108.

22. Hendriks ME, Wit FWNM, Roos MTL, et al. Hypertension in Sub-Saharan Africa: Cross-Sectional Surveys in Four Rural and Urban Communities. PLoS One **2012**; 7:e32638.

23. Hopkins KL, Hlongwane K, Otwombe K, et al. Demographics and health profile on precursors of non-communicable diseases in adults testing for HIV in Soweto, South Africa: A cross-sectional study. BMJ Open **2019**; 9:30701.

24. Jerico C, Knobel H, Montero M, et al. Hypertension in HIV-infected patients: prevalence and related factors. Am J Hypertens **2005**; 18:1396–1401.

25. Kavishe B, Biraro S, Baisley K, et al. High prevalence of hypertension and of risk factors for non-communicable diseases (NCDs): a population based cross-sectional survey of NCDS and HIV infection in Northwestern Tanzania and Southern Uganda. BMC Med **2015**; 13:126.

26. Kelly P, Katubulushi M, Todd J, et al. Micronutrient supplementation has limited effects on intestinal infectious disease and mortality in a Zambian population of mixed HIV status: a cluster randomized trial. Am J Clin Nutr **2008**; 88:1010–1017.

27. Kingery JR, Alfred Y, Smart LR, et al. Short-term and long-term cardiovascular risk, metabolic syndrome and HIV in Tanzania. Heart **2016**; 102:1200–1205.

28. Klein DB, Leyden WA, Xu L, et al. Declining Relative Risk for Myocardial Infarction Among HIV-Positive Compared With HIV-Negative Individuals With Access to Care. Clin Infect Dis **2015**; 60:1278–80.

29. Kunisaki KM, Akgun KM, Fiellin DA, et al. Prevalence and correlates of obstructive sleep apnoea among patients with and without HIV infection. HIV Med **2015**; 16:105–113.

30. Kwarisiima D, Balzer L, Heller D, et al. Population-Based Assessment of Hypertension Epidemiology and Risk Factors among HIV-Positive and General Populations in Rural Uganda. PLoS One **2016**; 11:e0156309.

31. Maciel RA, Kluck HM, Durand M, Sprinz E. Comorbidity is more common and occurs earlier in persons living with HIV than in HIV-uninfected matched controls, aged 50 years and older: A cross-sectional study. Int J Infect Dis **2018**; 70:30–35.

32. Malaza A, Mossong J, Barnighausen T, Newell M-L. Hypertension and obesity in adults living in a high HIV prevalence rural area in South Africa. PLoS One **2012**; 7:e47761.

33. Masyuko SJ, Page ST, Kinuthia J, et al. Metabolic syndrome and 10-year cardiovascular risk among HIV-positive and HIV-negative adults: A cross-sectional study. Medicine (Baltimore) **2020**; 99:e20845.

34. Mayer KH, Loo S, Crawford PM, et al. Excess clinical comorbidity among HIV-infected patients accessing primary care in US community health centers. Public Health Rep **2018**; 133:109–118.

35. Minami R, Takahama S, Yamamoto M. Correlates of telomere length shortening in peripheral leukocytes of HIV-infected individuals and association with leukoaraiosis. PLoS One **2019**; 14:e0218996.

36. Mondy K, Overton ET, Grubb J, et al. Metabolic syndrome in HIV-infected patients from an urban, midwestern US outpatient population. Clin Infect Dis **2007**; 44:726–734.

37. Monteiro P, Miranda-Filho DB, Bandeira F, et al. Is arterial stiffness in HIV-infected individuals associated with HIV-related factors? Brazilian J Med Biol Res **2012**; 45:818–826.

38. Mosha NR, Mahande M, Juma A, et al. Prevalence,awareness and factors associated with hypertension in North West Tanzania. Glob Health Action **2017**; 10:1321279.

39. Nakanga WP, Prynn JE, Banda L, et al. Prevalence of impaired renal function among rural and urban

populations: findings of a cross-sectional study in Malawi. Wellcome Open Res **2019**; 4.

40. Nakibuuka J, Sajatovic M, Nankabirwa J, et al. Stroke-Risk Factors Differ between Rural and Urban Communities: Population Survey in Central Uganda. Neuroepidemiology **2015**; 44:156–165.

41. Odden MC, Scherzer R, Bacchetti P, et al. Cystatin C level as a marker of kidney function in human immunodeficiency virus infection: the FRAM study. Arch Intern Med **2007**; 167:2213–2219.

42. Okello S, Ueda P, Kanyesigye M, et al. Association between HIV and blood pressure in adults and role of body weight as a mediator: Cross-sectional study in Uganda. J Clin Hypertens **2017**; 19:1181–1191.

43. Pacheco AG, Grinsztejn B, Fonseca M de JM da, et al. HIV infection is not associated with carotid intima-media thickness in Brazil: a cross-sectional analysis from the INI/ELSA-Brasil study. PLoS One **2016**; 11:e0158999.

44. Rucker SCM, Tayea A, Bitilinyu-Bangoh J, et al. High rates of hypertension, diabetes, elevated low-density lipoprotein cholesterol, and cardiovascular disease risk factors in HIV-infected patients in Malawi. AIDS **2018**; 32:253–260.

45. Russell E, Albert A, Côté H, et al. Rate of dyslipidemia higher among women living with HIV: A comparison of metabolic and cardiovascular health in a cohort to study aging in HIV. HIV Med **2020**; 21:418–428.

46. Ryscavage P, Still W, Nyemba V, Stafford K. Prevalence of Systemic Hypertension Among HIV-Infected and HIV-Uninfected Young Adults in Baltimore, Maryland. South Med J **2019**; 112:387–391.

47. Sanders J, Steverson A, Pawlowski A, et al. Atrial Fibrillation (AF) and Atrial Flutter (AFL) prevalence and characteristics for persons with Human Immunodeficiency Virus (HIV+) and matched uninfected controls. J Am Coll Cardiol **2017**; 69:540.

48. Sarfo FS, Nichols M, Agyei B, et al. Burden of subclinical carotid atherosclerosis and vascular risk factors among people living with HIV in Ghana. J Neurol Sci **2019**; 397:103–111.

49. Saves M, Chene G, Ducimetiere P, et al. Risk factors for coronary heart disease in patients treated for human immunodeficiency virus infection compared with the general population. Clin Infect Dis **2003**; 37:292–298.

50. Savinelli S, De Francesco D, Feeney E, et al. Factors associated with obesity in the Pharmacokinetic and Clinical Observations in People over Fifty (POPPY) cohort: an observational cross-sectional analysis. HIV Med **2020**; 21:441–452.

51. Scholten F, Mugisha J, Seeley J, et al. Health and functional status among older people with HIV/AIDS in Uganda. BMC Public Health **2011**; 11:886.

52. Schutte AE, Schutte R, Huisman HW, et al. Are behavioural risk factors to be blamed for the conversion from optimal blood pressure to hypertensive status in Black South Africans? A 5-year prospective study. Int J Epidemiol **2012**; 41:1114–1123.

53. Touloumi G, Kalpourtzi N, Papastamopoulos V, et al. Cardiovascular risk factors in HIV infected individuals: Comparison with general adult control population in Greece. PLoS One **2020**; 15.

54. Triant VA, Lee H, Hadigan C, Grinspoon SK. Increased Acute Myocardial Infarction Rates and Cardiovascular Risk Factors among Patients with Human Immunodeficiency Virus Disease. J Clin Endocrinol Metab **2007**; 92:2506–2512.

55. van Heerden A, Barnabas R V, Norris SA, Micklesfield LK, van Rooyen H, Celum C. High prevalence of HIV and non-communicable disease (NCD) risk factors in rural KwaZulu-Natal, South Africa. J Int AIDS Soc **2017**; 20:e25012.

56. van Zoest RA, Wit FW, Kooij KW, et al. Higher Prevalence of Hypertension in HIV-1-Infected Patients on Combination Antiretroviral Therapy Is Associated With Changes in Body Composition and Prior Stavudine Exposure. Clin Infect Dis **2016**; 63:205–213.

57. Watson C, Busovaca E, Foley JM, et al. White matter hyperintensities correlate to cognition and fiber tract integrity in older adults with HIV. J Neurovirol **2017**; 23:422–429.

58. Yang H-Y, Beymer MR, Suen S. Chronic Disease Onset Among People Living with HIV and AIDS in a Large Private Insurance Claims Dataset. Sci Rep **2019**; 9:18514.

59. Yu B, Pasipanodya E, Montoya JL, et al. Metabolic Syndrome and Neurocognitive Deficits in HIV Infection. J Acquir Immune Defic Syndr **2019**; 81:95–101.

Forest plot showing risk ratios by hypertension definition

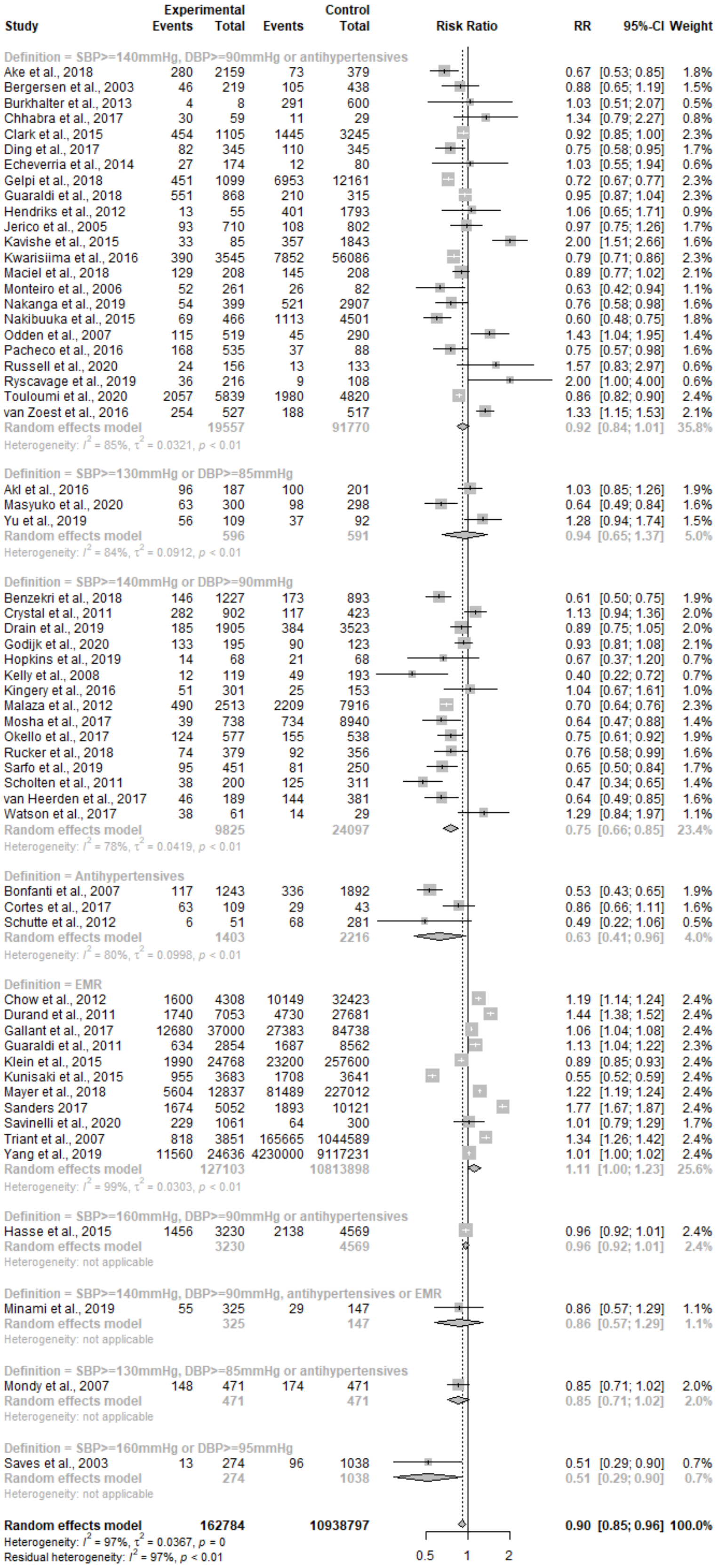

Supplementary figure 1. **Forest plot of the risk ratio for hypertension by HIV status, categorised by hypertension definition. Estimates were pooled using a random effects model, with a p-value for sub-group differences of 0.0001. SBP: Systolic Blood Pressure, DBP: Diastolic Blood Pressure, EMR: Electronic Medical Records.**

Univariate meta-regression results

Supplementary table 1. **Univariate meta-regressions.**

**Variable**	**Number of studies**	**Number of participants**	**Risk ratio (95% confidence interval)**	**P-value for sub-category**	**Overall p-value**	R^2
Hypertension definition					**0.004**	15.49%
SBP≥140mmHg and/or DBP≥90mmHg and/or antidepressant use	41	148,868	1	-		
EMR	11	10,941,001	1.33 (1.16-1.51)	**<0.001**		
Other	7	11,712	1.06 (0.89-1.27)	0.515		
Continent					**<0.001**	15.17%
Africa	22	112,005	1	-		
Asia	2	1,162	1.04 (0.74-1.47)	0.806		
Europe	13	53,680	1.21 (1.04-1.40)	**0.012**		
North America	18	10,932,964	1.49 (1.30-1.70)	**<0.001**		
South America	4	1,770	1.12 (0.89-1.41)	0.346		
Year study began					0.366	<0.00%
2005 onwards	43	9,663,958	1	-		
Before 2005	10	1,375,532	0.95 (0.81-1.11)	0.520		
Not reported	6	62,091	1.03 (0.84-1.27)	0.756		
Mean age (years) *	32	9,919,853	1.00 (0.99-1.01)	-	0.705	<0.00%
Proportion on ART (%) *	40	1,595,861	1.30 (0.92-1.83)	-	0.130	<0.00%
Proportion female (%) *	56	1,945,979	1.00 (0.99-1.00)	-	0.994	0.52%

SBP: Systolic Blood Pressure, DBP: Diastolic Blood Pressure, EMR: Electronic Medical Records.

* Where variables were examined using a continuous scale, studies without data were dropped from the analysis.

We also saw no effect of study start time on the risk ratio. This remained the case if start time was considered as a continuous variable and may reflect the fact that the study length was highly variable, with some studies running for many years and others ending in the early days of improved ART access.

Age and gender are known to be associated with hypertension, but we did not find a univariate association between either of these variables and the risk ratio in the meta-regression. Categorising mean age or using median age or each study's age limits instead of mean age, did not change these conclusions. We expect this occurred because the two variables had a similar effect among PLHIV and HIV-negative individuals, resulting in minimal change to the risk ratio if they were altered.

Finally, we found no effect of the proportion of PLHIV on ART on relationship between HIV and hypertension in univariate analyses. This was unexpected as previous meta-analyses have demonstrated an increase in hypertension associated with ART usage. The lack of a relationship may reflect heterogeneity in availability of ART and reporting of ART usage between studies.

Permutation test results

***Supplementary table 2*. Multivariable meta-regression from 1,000 permutations of the data.**

**Variable**	**Risk ratio (95% confidence interval)**	**P-value**
Hypertension definition		
Includes SBP≥140mmHg and/or DBP≥90mmHg and/or antihypertensive use	1	-
EMR	1.05 (0.84-1.32)	0.700
Other	0.99 (0.78-1.26)	0.783
Continent		
Africa	1	-
Asia	1.06 (0.71-1.59)	0.775
Europe	1.21 (0.99-1.47)	0.087
North America	1.46 (1.16-1.83)	**0.004**
South America	1.12 (0.84-1.48)	0.434

SBP: Systolic Blood Pressure, DBP: Diastolic Blood Pressure, EMR: Electronic Medical Records.

After controlling for study location, we saw no effect of hypertension definition on the risk ratio. It appears likely that the univariate association was the result of an association between study continent and hypertension definition used. This implies that the use of different definitions did not affect the main result, giving confidence that our results are robust.

Risk of bias assessment

Supplementary table 3. **Risk of bias assessment for included studies.**

First author, year published	Was the research question or objective clearly stated?	Was the study population clearly specified and defined?	Was the participation rate of eligible persons at least 50%?	Were all subjects recruited from the same or similar populations (including the same time period)?	Were inclusion/ exclusion criteria prespecified and applied uniformly to all participants?	Was a sample size justification, power description, or variance and effect estimates provided?	Were the exposure measures (independent variables) clearly defined, valid, reliable, and implemented consistently across all study participants?	Were the outcome measures (dependent variables) clearly defined, valid, reliable, and implemented consistently across all study participants?	Were the outcome assessors blinded to the exposure status of participants?	Were the exposure(s) of interest measured prior to the outcome(s) being measured?	Risk of bias
Ake, 2019	Yes	Yes	NR	No	Yes	No	Yes	Yes	NR	Yes	Medium
Akl, 2016	Yes	Yes	NR	No	Yes	Yes	Yes	Yes	NR	Yes	Low
Benzekri, 2018	Yes	Yes	NR	No	No	No	Yes	Yes	NR	NR	High
Bergersen, 2003	Yes	Yes	No	Yes	Yes	No	Yes	Yes	NR	Yes	Medium
Bonfanti, 2007	Yes	Yes	NR	No	No	No	Yes	Yes	NR	Yes	High
Burkhalter, 2013	Yes	Yes	Yes	Yes	Yes	No	Yes	Yes	NR	NR	Low
Chhabra, 2017	Yes	Yes	NR	Yes	Yes	No	NR	Yes	NR	Yes	Low
Chow, 2012	Yes	Yes	NA	Yes	Yes	No	Yes	Yes	NR	NR	Low
Clark, 2015	Yes	Yes	Yes	No	Yes	No	Yes	Yes	NR	NR	Medium
Cortes, 2017	Yes	Yes	NR	No	Yes	No	Yes	Yes	NR	NR	Medium
Crystal, 2011	Yes	Yes	Yes	No	Yes	No	Yes	Yes	NR	Yes	Medium
Ding, 2017	Yes	Yes	NR	Yes	Yes	No	Yes	Yes	NR	Yes	Low
Drain, 2019	Yes	Yes	NR	Yes	Yes	No	Yes	Yes	Yes	No	Low
Durand, 2011	Yes	Yes	NA	Yes	Yes	No	Yes	Yes	NR	NR	Low
Echeverria, 2014	Yes	Yes	Yes	No	Yes	Yes	Yes	Yes	NR	Yes	Low
Gallant, 2017	Yes	Yes	NA	Yes	Yes	No	Yes	Yes	NR	NR	Low
Gelpi, 2018	Yes	Yes	No	Yes	Yes	No	Yes	Yes	NR	Yes	Medium

Godijk, 2020	Yes	Yes	Yes	Yes	NR	Yes	Yes	Yes	NR	NR	Low
Guaraldi, 2011	Yes	Yes	NR	No	Yes	No	Yes	Yes	NR	NR	Medium
Guaraldi, 2018	Yes	Yes	NR	No	Yes	No	Yes	Yes	NR	NR	Medium
Hasse, 2015	Yes	Yes	NR	No	Yes	No	Yes	Yes	NR	NR	Medium
Hendriks, 2012	Yes	Yes	Yes	Yes	Yes	No	Yes	Yes	NR	NR	Low
Hopkins, 2020	Yes	Yes	NR	Yes	Yes	No	Yes	Yes	Yes	Yes	Low
Jerico, 2005	Yes	Yes	Yes	No	Yes	No	Yes	Yes	Yes	Yes	Medium
Kavishe, 2015	Yes	Yes	Yes	Yes	Yes	Yes	Yes	Yes	NR	NR	Low
Kelly, 2008	Yes	Yes	Yes	Yes	Yes	Yes	Yes	Yes	NR	NR	Low
Kingery, 2016	Yes	Yes	Yes	No	Yes	Yes	Yes	Yes	NR	Yes	Low
Klein, 2015	Yes	Yes	NA	Yes	Yes	No	Yes	Yes	NR	NR	Low
Kunisaki, 2014	Yes	Yes	NR	Yes	Yes	No	Yes	Yes	NR	Yes	Low
Kwarisiima, 2016	Yes	Yes	Yes	Yes	Yes	No	Yes	Yes	NR	NR	Low
Maciel, 2018	Yes	Yes	Yes	No	Yes	Yes	Yes	Yes	NR	NR	Low
Malaza, 2012	Yes	Yes	Yes	Yes	Yes	No	Yes	Yes	NR	NR	Low
Masyuko, 2020	Yes	Yes	NR	No	Yes	No	Yes	Yes	NR	NR	Medium
Mayer, 2018	Yes	Yes	NA	Yes	Yes	No	Yes	Yes	NR	NR	Low
Minami, 2019	Yes	Yes	NR	No	Yes	No	Yes	Yes	NR	NR	Low
Mondy, 2006	Yes	Yes	Yes	No	Yes	No	Yes	Yes	NR	NR	Low
Monteiro, 2006	Yes	Yes	NR	No	Yes	No	Yes	Yes	NR	Yes	Medium
Mosha, 2017	Yes	Yes	Yes	Yes	Yes	Yes	Yes	Yes	Yes	Yes	Low
Nakanga, 2019	Yes	Yes	Yes	No	Yes	Yes	Yes	Yes	NR	NR	Low
Nakibuuka, 2015	Yes	Yes	NR	Yes	Yes	No	Yes	Yes	NR	NR	Low
Odden, 2007	Yes	Yes	NR	No	Yes	No	Yes	Yes	NR	NR	Medium
Okello, 2017	Yes	Yes	Yes	Yes	Yes	No	Yes	Yes	NR	Yes	Low

Pacheco, 2016	Yes	Yes	Yes	No	NR	No	Yes	Yes	NR	Yes	Medium
Rucker, 2018	Yes	Yes	Yes	No	Yes	No	Yes	Yes	NR	NR	Medium
Russell, 2020	Yes	Yes	NR	No	Yes	No	Yes	Yes	NR	NR	Medium
Ryscavage, 2019	Yes	Yes	NR	Yes	Yes	No	Yes	Yes	NR	NR	Low
Sanders 2017	Yes	Yes	NR	Yes	Yes	No	Yes	Yes	NR	NR	Low
Sarfo, 2019	Yes	Yes	NR	Yes	Yes	No	Yes	Yes	NR	Yes	Low
Saves, 2003	Yes	Yes	NR	No	NR	No	Yes	Yes	NR	Yes	Medium
Savinelli, 2020	Yes	Yes	Yes	No	Yes	Yes	Yes	Yes	NR	Yes	Low
Scholten, 2011	Yes	Yes	Yes	Yes	No	No	Yes	Yes	NR	Yes	Medium
Schutte, 2012	Yes	Yes	NR	Yes	Yes	No	Yes	Yes	NR	NR	Low
Touloumi, 2020	Yes	Yes	Yes	No	Yes	No	Yes	Yes	NR	Yes	Medium
Triant, 2007	Yes	Yes	NA	Yes	Yes	No	Yes	Yes	NR	NR	Low
van Heerden, 2017	Yes	Yes	Yes	Yes	NR	No	Yes	Yes	NR	NR	Low
van Zoest, 2016	Yes	Yes	Yes	No	Yes	No	Yes	Yes	NR	Yes	Medium
Watson, 2017	Yes	Yes	NR	No	Yes	No	Yes	Yes	NR	Yes	Medium
Yang, 2020	Yes	Yes	NA	Yes	Yes	No	Yes	Yes	NR	Yes	Low
Yu, 2019	Yes	Yes	NR	No	Yes	No	Yes	Yes	NR	Yes	Medium

NR: Not Reported; NA: Not Applicable.

Several questions were not included in quality assessment as they were not relevant for these studies, which were cross-sectional analyses of a binary outcome and used only unadjusted results. The questions which were removed are:

- For exposures that can vary in amount or level, did the study examine different levels of the exposure as related to the outcome (e.g., categories of exposure, or exposure measured as continuous variable)?
- Was the exposure(s) assessed more than once over time?
- Were key potential confounding variables measured and adjusted statistically for their impact on the relationship between exposure(s) and outcome(s)?
- Was the timeframe sufficient so that one could reasonably expect to see an association between exposure and outcome if it existed?
- Was loss to follow-up after baseline 20% or less?

Results with high-risk studies removed

Study	Experimental Events	Experimental Total	Control Events	Control Total	RR	95%-CI	Weight
**Continent = Africa**							
Ake et al., 2018	280	2159	73	379	0.67	[0.53; 0.85]	1.8%
Clark et al., 2015	454	1105	1445	3245	0.92	[0.85; 1.00]	2.4%
Drain et al., 2019	185	1905	384	3523	0.89	[0.75; 1.05]	2.1%
Godijk et al., 2020	133	195	90	123	0.93	[0.81; 1.08]	2.2%
Hendriks et al., 2012	13	55	401	1793	1.06	[0.65; 1.71]	0.9%
Hopkins et al., 2019	14	68	21	68	0.67	[0.37; 1.20]	0.7%
Kavishe et al., 2015	33	85	357	1843	2.00	[1.51; 2.66]	1.6%
Kelly et al., 2008	12	119	49	193	0.40	[0.22; 0.72]	0.7%
Kingery et al., 2016	51	301	25	153	1.04	[0.67; 1.61]	1.1%
Kwarisiima et al., 2016	390	3545	7852	56086	0.79	[0.71; 0.86]	2.4%
Malaza et al., 2012	490	2513	2209	7916	0.70	[0.64; 0.76]	2.4%
Masyuko et al., 2020	63	300	98	298	0.64	[0.49; 0.84]	1.6%
Mosha et al., 2017	39	738	734	8940	0.64	[0.47; 0.88]	1.5%
Nakanga et al., 2019	54	399	521	2907	0.76	[0.58; 0.98]	1.7%
Nakibuuka et al., 2015	69	466	1113	4501	0.60	[0.48; 0.75]	1.9%
Okello et al., 2017	124	577	155	538	0.75	[0.61; 0.92]	1.9%
Rucker et al., 2018	74	379	92	356	0.76	[0.58; 0.99]	1.7%
Sarfo et al., 2019	95	451	81	250	0.65	[0.50; 0.84]	1.7%
Scholten et al., 2011	38	200	125	311	0.47	[0.34; 0.65]	1.5%
Schutte et al., 2012	6	51	68	281	0.49	[0.22; 1.06]	0.5%
van Heerden et al., 2017	46	189	144	381	0.64	[0.49; 0.85]	1.6%
Random effects model		15800		94085	0.76	[0.68; 0.85]	34.0%
Heterogeneity: $I^2 = 82\%$, $\tau^2 = 0.0408$, $p < 0.01$							
**Continent = South America**							
Akl et al., 2016	96	187	100	201	1.03	[0.85; 1.26]	2.0%
Maciel et al., 2018	129	208	145	208	0.89	[0.77; 1.02]	2.2%
Monteiro et al., 2006	52	261	26	82	0.63	[0.42; 0.94]	1.2%
Pacheco et al., 2016	168	535	37	88	0.75	[0.57; 0.98]	1.6%
Random effects model		1191		579	0.85	[0.72; 1.01]	7.0%
Heterogeneity: $I^2 = 55\%$, $\tau^2 = 0.0158$, $p = 0.08$							
**Continent = Europe**							
Bergersen et al., 2003	46	219	105	438	0.88	[0.65; 1.19]	1.5%
Chhabra et al., 2017	30	59	11	29	1.34	[0.79; 2.27]	0.8%
Echeverria et al., 2014	27	174	12	80	1.03	[0.55; 1.94]	0.7%
Gelpi et al., 2018	451	1099	6953	12161	0.72	[0.67; 0.77]	2.5%
Guaraldi et al., 2011	634	2854	1687	8562	1.13	[1.04; 1.22]	2.4%
Guaraldi et al., 2018	551	868	210	315	0.95	[0.87; 1.04]	2.4%
Hasse et al., 2015	1456	3230	2138	4569	0.96	[0.92; 1.01]	2.5%
Jerico et al., 2005	93	710	108	802	0.97	[0.75; 1.26]	1.7%
Saves et al., 2003	13	274	96	1038	0.51	[0.29; 0.90]	0.8%
Savinelli et al., 2020	229	1061	64	300	1.01	[0.79; 1.29]	1.8%
Touloumi et al., 2020	2057	5839	1980	4820	0.86	[0.82; 0.90]	2.5%
van Zoest et al., 2016	254	527	188	517	1.33	[1.15; 1.53]	2.2%
Random effects model		16914		33631	0.96	[0.86; 1.07]	21.7%
Heterogeneity: $I^2 = 90\%$, $\tau^2 = 0.0243$, $p < 0.01$							
**Continent = North America**							
Burkhalter et al., 2013	4	8	291	600	1.03	[0.51; 2.07]	0.6%
Chow et al., 2012	1600	4308	10149	32423	1.19	[1.14; 1.24]	2.5%
Cortes et al., 2017	63	109	29	43	0.86	[0.66; 1.11]	1.7%
Crystal et al., 2011	282	902	117	423	1.13	[0.94; 1.36]	2.1%
Durand et al., 2011	1740	7053	4730	27681	1.44	[1.38; 1.52]	2.5%
Gallant et al., 2017	12680	37000	27383	84738	1.06	[1.04; 1.08]	2.5%
Klein et al., 2015	1990	24768	23200	257600	0.89	[0.85; 0.93]	2.5%
Kunisaki et al., 2015	955	3683	1708	3641	0.55	[0.52; 0.59]	2.5%
Mayer et al., 2018	5604	12837	81489	227012	1.22	[1.19; 1.24]	2.5%
Mondy et al., 2007	148	471	174	471	0.85	[0.71; 1.02]	2.1%
Odden et al., 2007	115	519	45	290	1.43	[1.04; 1.95]	1.5%
Russell et al., 2020	24	156	13	133	1.57	[0.83; 2.97]	0.6%
Ryscavage et al., 2019	36	216	9	108	2.00	[1.00; 4.00]	0.6%
Sanders 2017	1674	5052	1893	10121	1.77	[1.67; 1.87]	2.5%
Triant et al., 2007	818	3851	165665	1044589	1.34	[1.26; 1.42]	2.5%
Watson et al., 2017	38	61	14	29	1.29	[0.84; 1.97]	1.1%
Yang et al., 2019	11560	24636	4230000	9117231	1.01	[1.00; 1.02]	2.5%
Yu et al., 2019	56	109	37	92	1.28	[0.94; 1.74]	1.5%
Random effects model		125739		10807225	1.12	[1.02; 1.23]	34.3%
Heterogeneity: $I^2 = 99\%$, $\tau^2 = 0.0308$, $p < 0.01$							
**Continent = Asia**							
Ding et al., 2017	82	345	110	345	0.75	[0.58; 0.95]	1.8%
Minami et al., 2019	55	325	29	147	0.86	[0.57; 1.29]	1.2%
Random effects model		670		492	0.77	[0.63; 0.95]	2.9%
Heterogeneity: $I^2 = 0\%$, $\tau^2 = 0$, $p = 0.56$							
**Random effects model**		**160314**		**10936012**	**0.92**	**[0.87; 0.98]**	**100.0%**
Heterogeneity: $I^2 = 97\%$, $\tau^2 = 0.0355$, $p = 0$							
Residual heterogeneity: $I^2 = 96\%$, $p < 0.01$							

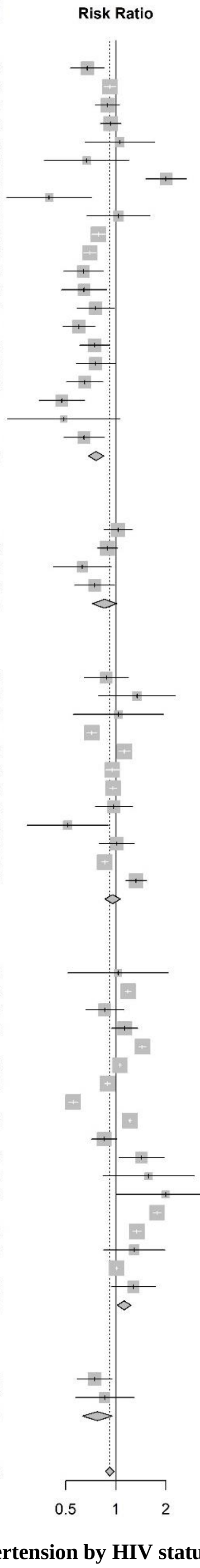

Supplementary figure 2. **Forest plot of the risk ratio for hypertension by HIV status, categorised by continent, with the two high-risk studies removed. Estimates were pooled using a random effects model, with a p-value for sub-group differences of less than 0.0001.**

Supplementary table 4. **Multivariable meta-regression without high-risk studies (R^2=12.19%).**

**Variable**	**Number of studies**	**Number of participants**	**Risk ratio (95% confidence interval)**	**P-value for sub-category**	**Overall p-value**
Hypertension definition					0.638
Includes SBP≥140mmHg and/or DBP≥90mmHg and/or antihypertensive use	39	143,613	1	-	
EMR	11	1,0941,001	1.02 (0.85-1.21)	0.856	
Other	7	11,712	0.94 (0.78-1.14)	0.551	
Continent					**0.001**
Africa	21	109,885	1	-	
Asia	2	1,162	1.05 (0.74-1.49)	0.777	
Europe	12	50,545	1.26 (1.07-1.48)	**0.005**	
North America	18	10,932,964	1.46 (1.21-1.75)	**<0.001**	
South America	4	1,770	1.12 (0.88-1.42)	0.349	

SBP: Systolic Blood Pressure, DBP: Diastolic Blood Pressure, EMR: Electronic Medical Records.

Contour-enhanced funnel plots and Egger's test results

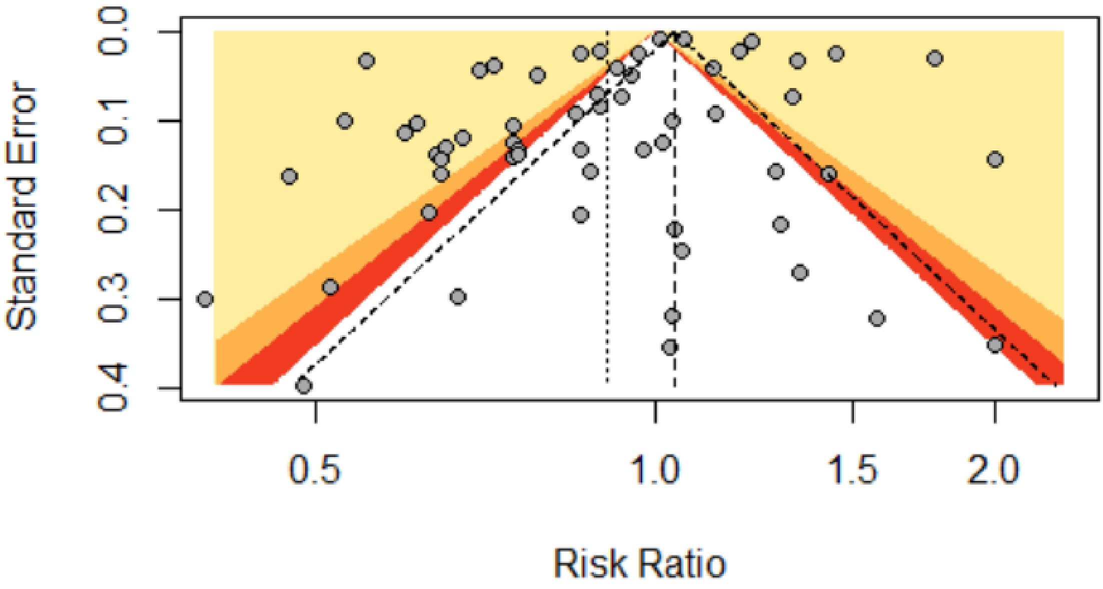

Supplementary figure 3. **Contour-enhanced funnel plot with all studies. Red: p<0.05, orange: p<0.025, yellow: p<0.01.**

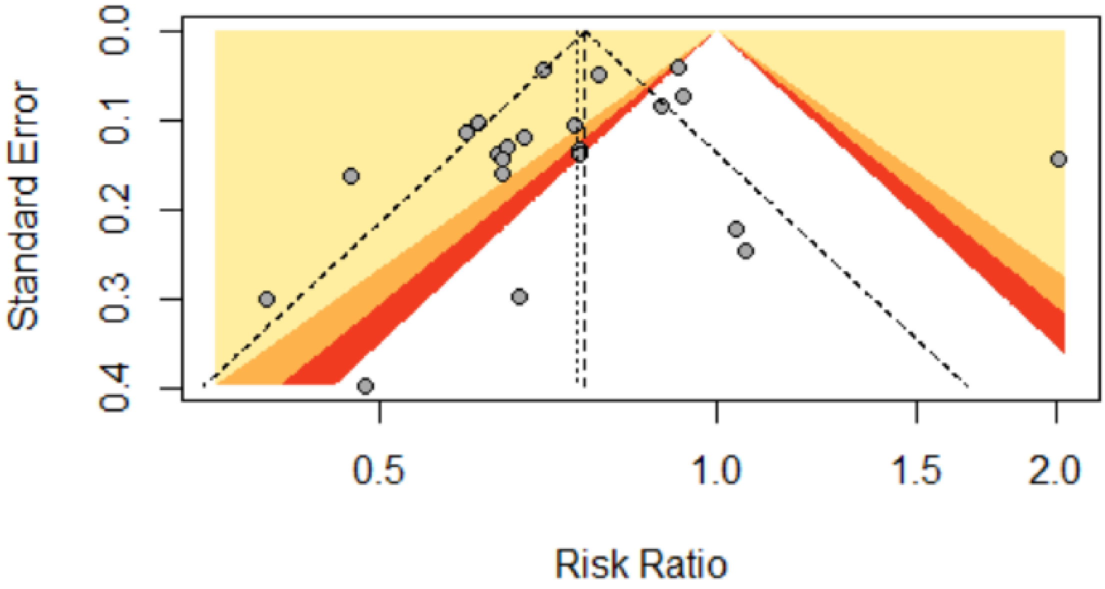

Supplementary figure 4. **Contour-enhanced funnel plot with studies from Africa. Red: p<0.05, orange: p<0.025, yellow: p<0.01.**

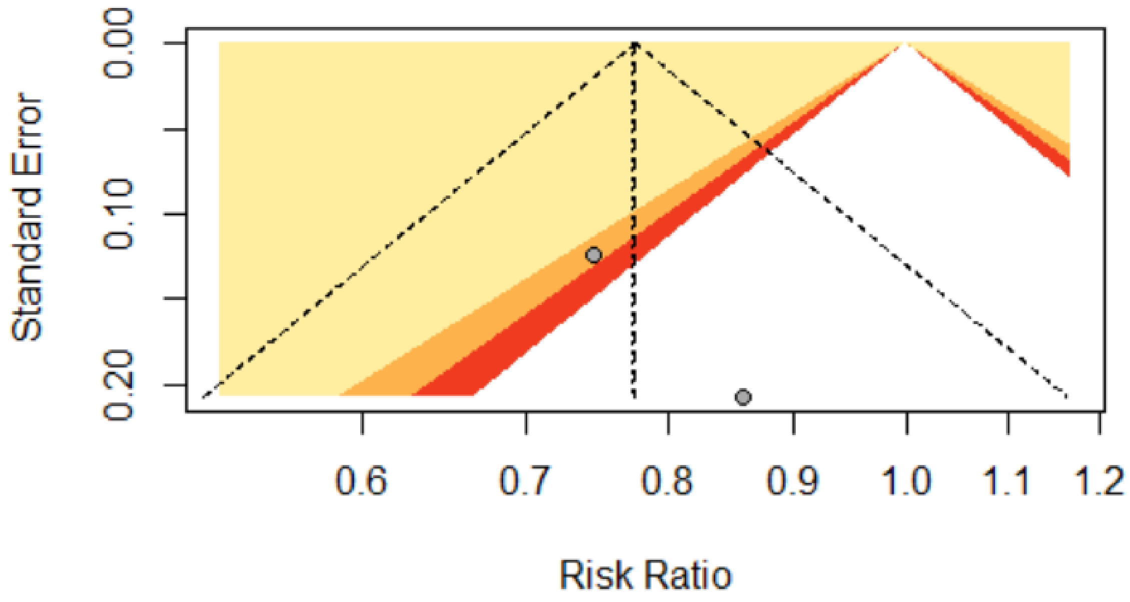

Supplementary figure 5. **Contour-enhanced funnel plot with studies from Asia. Red: p<0.05, orange: p<0.025, yellow: p<0.01.**

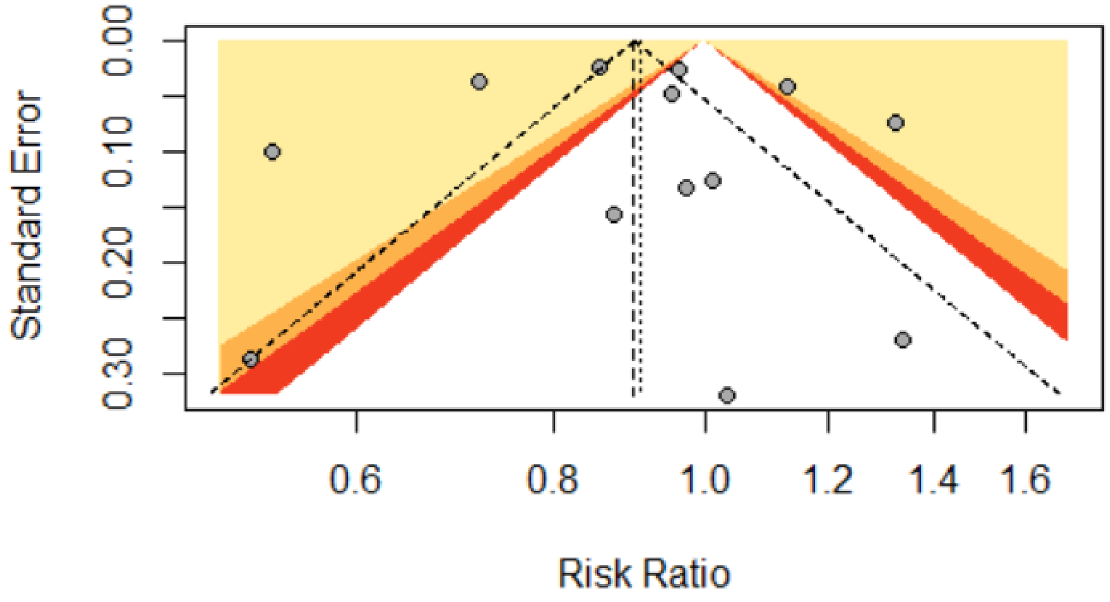

Supplementary figure 6. **Contour-enhanced funnel plot with studies from Europe. Red: p<0.05, orange: p<0.025, yellow: p<0.01.**

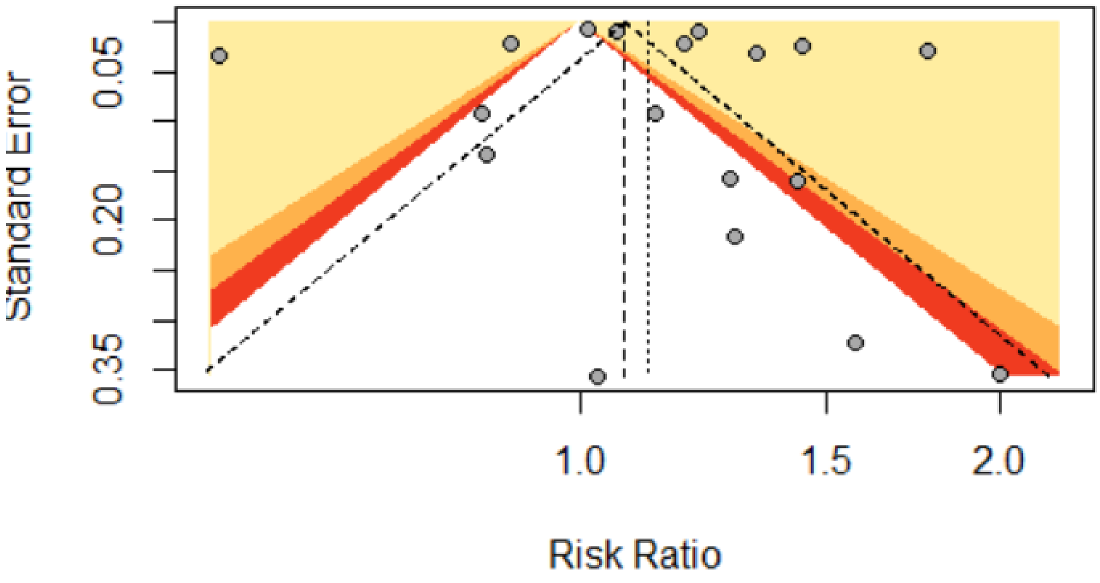

Supplementary figure 7. **Contour-enhanced funnel plot with studies from North America. Red: p<0.05, orange: p<0.025, yellow: p<0.01.**

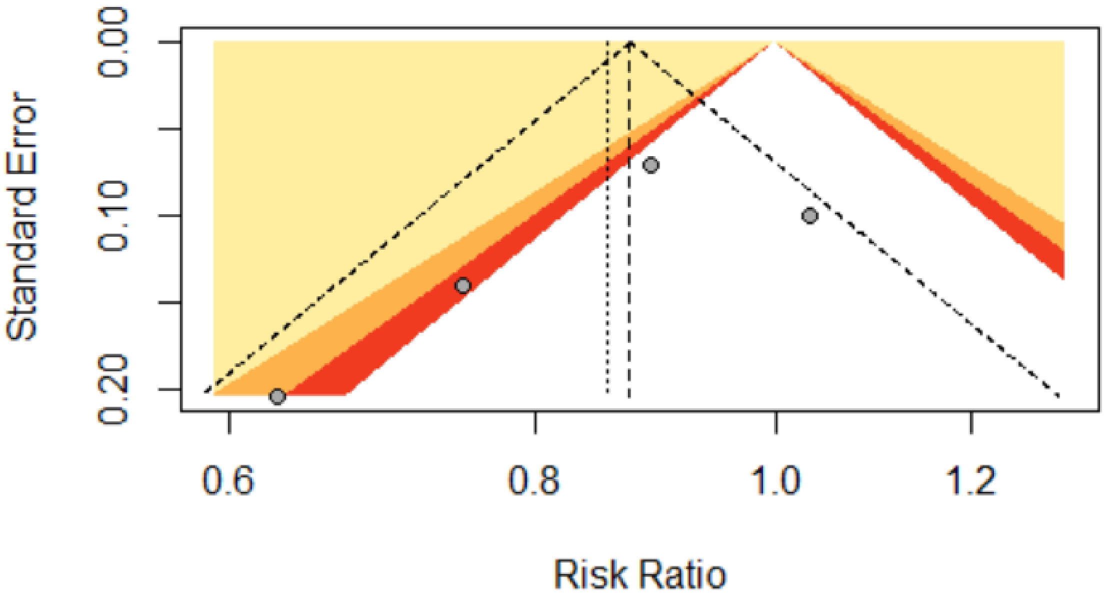

Supplementary figure 8. **Contour-enhanced funnel plot with studies from South America. Red: p<0.05, orange: p<0.025, yellow: p<0.01.**

***Supplementary table 5.* Egger's test results.**

**Studies included**	**Intercept**	**P-value**
All studied	0.07	**0.049**
African studies	-0.16	0.294
European studies	-0.10	0.939
North American studies	0.06	0.612

Results with Hartung-Knapp modification

Study	Experimental Events	Experimental Total	Control Events	Control Total	RR	95%-CI	Weight
**Continent = Africa**							
Ake et al., 2018	280	2159	73	379	0.67	[0.53; 0.85]	1.8%
Benzekri et al., 2018	146	1227	173	893	0.61	[0.50; 0.75]	1.9%
Clark et al., 2015	454	1105	1445	3245	0.92	[0.85; 1.00]	2.3%
Drain et al., 2019	185	1905	384	3523	0.89	[0.75; 1.05]	2.0%
Godijk et al., 2020	133	195	90	123	0.93	[0.81; 1.08]	2.1%
Hendriks et al., 2012	13	55	401	1793	1.06	[0.65; 1.71]	0.9%
Hopkins et al., 2019	14	68	21	68	0.67	[0.37; 1.20]	0.7%
Kavishe et al., 2015	33	85	357	1843	2.00	[1.51; 2.66]	1.6%
Kelly et al., 2008	12	119	49	193	0.40	[0.22; 0.72]	0.7%
Kingery et al., 2016	51	301	25	153	1.04	[0.67; 1.61]	1.0%
Kwarisiima et al., 2016	390	3545	7852	56086	0.79	[0.71; 0.86]	2.3%
Malaza et al., 2012	490	2513	2209	7916	0.70	[0.64; 0.76]	2.3%
Masyuko et al., 2020	63	300	98	298	0.64	[0.49; 0.84]	1.6%
Mosha et al., 2017	39	738	734	8940	0.64	[0.47; 0.88]	1.4%
Nakanga et al., 2019	54	399	521	2907	0.76	[0.58; 0.98]	1.6%
Nakibuuka et al., 2015	69	466	1113	4501	0.60	[0.48; 0.75]	1.8%
Okello et al., 2017	124	577	155	538	0.75	[0.61; 0.92]	1.9%
Rucker et al., 2018	74	379	92	356	0.76	[0.58; 0.99]	1.6%
Sarfo et al., 2019	95	451	81	250	0.65	[0.50; 0.84]	1.7%
Scholten et al., 2011	38	200	125	311	0.47	[0.34; 0.65]	1.4%
Schutte et al., 2012	6	51	68	281	0.49	[0.22; 1.06]	0.5%
van Heerden et al., 2017	46	189	144	381	0.64	[0.49; 0.85]	1.6%
Random effects model		17027		94978	0.75	[0.68; 0.83]	34.7%
Heterogeneity: $I^2 = 82\%$, $\tau^2 = 0.0412$, $p < 0.01$							
**Continent = South America**							
Akl et al., 2016	96	187	100	201	1.03	[0.85; 1.26]	1.9%
Maciel et al., 2018	129	208	145	208	0.89	[0.77; 1.02]	2.1%
Monteiro et al., 2006	52	261	26	82	0.63	[0.42; 0.94]	1.1%
Pacheco et al., 2016	168	535	37	88	0.75	[0.57; 0.98]	1.6%
Random effects model		1191		579	0.85	[0.72; 1.01]	6.8%
Heterogeneity: $I^2 = 55\%$, $\tau^2 = 0.0158$, $p = 0.08$							
**Continent = Europe**							
Bergersen et al., 2003	46	219	105	438	0.88	[0.65; 1.19]	1.5%
Bonfanti et al., 2007	117	1243	336	1892	0.53	[0.43; 0.65]	1.9%
Chhabra et al., 2017	30	59	11	29	1.34	[0.79; 2.27]	0.8%
Echeverria et al., 2014	27	174	12	80	1.03	[0.55; 1.94]	0.6%
Gelpi et al., 2018	451	1099	6953	12161	0.72	[0.67; 0.77]	2.3%
Guaraldi et al., 2011	634	2854	1687	8562	1.13	[1.04; 1.22]	2.3%
Guaraldi et al., 2018	551	868	210	315	0.95	[0.87; 1.04]	2.3%
Hasse et al., 2015	1456	3230	2138	4569	0.96	[0.92; 1.01]	2.4%
Jerico et al., 2005	93	710	108	802	0.97	[0.75; 1.26]	1.7%
Saves et al., 2003	13	274	96	1038	0.51	[0.29; 0.90]	0.7%
Savinelli et al., 2020	229	1061	64	300	1.01	[0.79; 1.29]	1.7%
Touloumi et al., 2020	2057	5839	1980	4820	0.86	[0.82; 0.90]	2.4%
van Zoest et al., 2016	254	527	188	517	1.33	[1.15; 1.53]	2.1%
Random effects model		18157		35523	0.91	[0.81; 1.02]	22.8%
Heterogeneity: $I^2 = 92\%$, $\tau^2 = 0.0302$, $p < 0.01$							
**Continent = North America**							
Burkhalter et al., 2013	4	8	291	600	1.03	[0.51; 2.07]	0.5%
Chow et al., 2012	1600	4308	10149	32423	1.19	[1.14; 1.24]	2.4%
Cortes et al., 2017	63	109	29	43	0.86	[0.66; 1.11]	1.6%
Crystal et al., 2011	282	902	117	423	1.13	[0.94; 1.36]	2.0%
Durand et al., 2011	1740	7053	4730	27681	1.44	[1.38; 1.52]	2.4%
Gallant et al., 2017	12680	37000	27383	84738	1.06	[1.04; 1.08]	2.4%
Klein et al., 2015	1990	24768	23200	257600	0.89	[0.85; 0.93]	2.4%
Kunisaki et al., 2015	955	3683	1708	3641	0.55	[0.52; 0.59]	2.4%
Mayer et al., 2018	5604	12837	81489	227012	1.22	[1.19; 1.24]	2.4%
Mondy et al., 2007	148	471	174	471	0.85	[0.71; 1.02]	2.0%
Odden et al., 2007	115	519	45	290	1.43	[1.04; 1.95]	1.4%
Russell et al., 2020	24	156	13	133	1.57	[0.83; 2.97]	0.6%
Ryscavage et al., 2019	36	216	9	108	2.00	[1.00; 4.00]	0.6%
Sanders 2017	1674	5052	1893	10121	1.77	[1.67; 1.87]	2.4%
Triant et al., 2007	818	3851	165665	1044589	1.34	[1.26; 1.42]	2.4%
Watson et al., 2017	38	61	14	29	1.29	[0.84; 1.97]	1.1%
Yang et al., 2019	11560	24636	4230000	9117231	1.01	[1.00; 1.02]	2.4%
Yu et al., 2019	56	109	37	92	1.28	[0.94; 1.74]	1.5%
Random effects model		125739		10807225	1.12	[1.02; 1.23]	32.9%
Heterogeneity: $I^2 = 99\%$, $\tau^2 = 0.0308$, $p < 0.01$							
**Continent = Asia**							
Ding et al., 2017	82	345	110	345	0.75	[0.58; 0.95]	1.7%
Minami et al., 2019	55	325	29	147	0.86	[0.57; 1.29]	1.1%
Random effects model		670		492	0.77	[0.63; 0.95]	2.8%
Heterogeneity: $I^2 = 0\%$, $\tau^2 = 0$, $p = 0.56$							
**Random effects model**		**162784**		**10938797**	**0.90**	**[0.85; 0.96]**	**100.0%**
Heterogeneity: $I^2 = 97\%$, $\tau^2 = 0.0367$, $p = 0$							
Residual heterogeneity: $I^2 = 96\%$, $p < 0.01$							

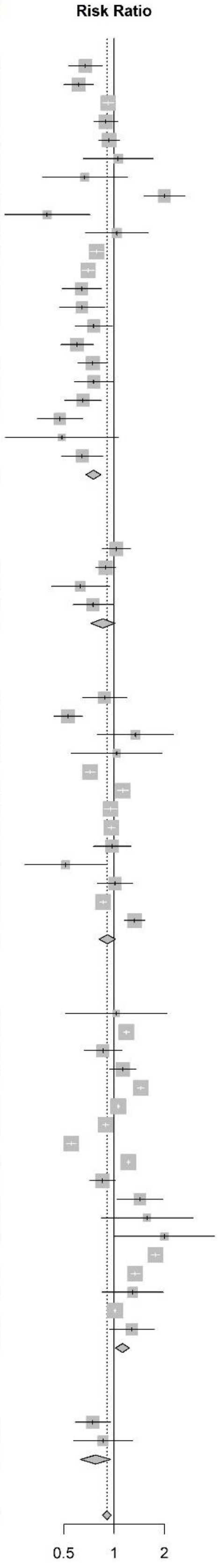

Supplementary figure 9. **Forest plot of the risk ratio for hypertension by HIV status, categorised by continent, with the Hartung-Knapp modification. Estimates were pooled using a random effects model, with a p-value for sub-group differences of 0.0002.**

Supplementary table 6. **Multivariable meta-regression for meta-analyses with the Hartung-Knapp modification (R^2=15.17%).**

**Variable**	**Number of studies**	**Number of participants**	**Risk ratio (95% confidence interval)**	**P-value for sub-category**	**Overall p-value**
Hypertension definition					**0.002**
Includes SBP≥140mmHg and/or DBP≥90mmHg and/or antihypertensive use	41	148,868	1	-	
EMR	11	10,941,001	1.04 (0.82-1.33)	0.730	
Other	7	11,712	0.97 (0.75-1.26)	0.810	
Continent					0.057
Africa	22	112,005	1	-	
Asia	2	1,162	1.06 (0.65-1.71)	0.823	
Europe	13	53,680	1.20 (0.97-1.49)	0.085	
North America	18	10,932,964	1.45 (1.13-1.86)	**0.004**	
South America	4	1,770	1.13 (0.81-1.56)	0.467	

SBP: Systolic Blood Pressure, DBP: Diastolic Blood Pressure, EMR: Electronic Medical Records.

UNAIDS regions sensitivity analysis

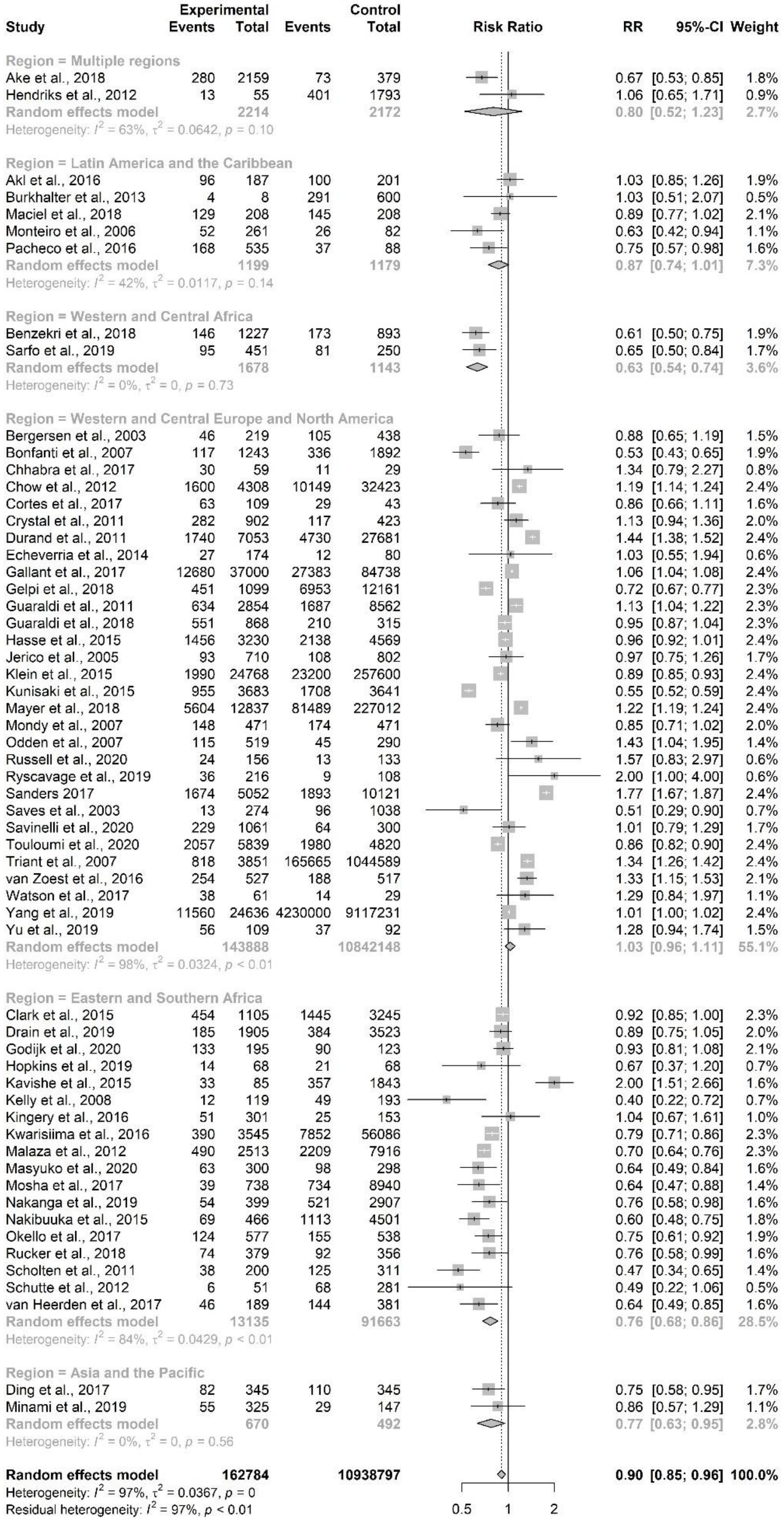

***Supplementary figure 10*. Forest plot of the risk ratio for hypertension by HIV status, categorised by UNAIDS region. Estimates were pooled using a random effects model, with a p-value for sub-group differences of <0.0001.**
